# Effects of low-doses of methamphetamine on *d*-fenfluramine-induced head-twitch response (HTR) in mice during ageing and c-*fos* expression in the prefrontal cortex

**DOI:** 10.1186/s12868-022-00766-0

**Published:** 2023-01-11

**Authors:** Yina Sun, Seetha Chebolu, Stone Skegrud, Setareh Kamali, Nissar A. Darmani

**Affiliations:** Department of Basic Medical Sciences, College of Osteopathic Medicine of the Pacific, Western University of Health Sciences, 309 East Second Street, Pomona, CA 91766 USA

**Keywords:** Methamphetamine, *d*-fenfluramine, Head-twitch response, 5-HT_2A_ receptor, 5-HT_1A_ receptor, Adrenergic ɑ_2_-receptors

## Abstract

**Background:**

The head-twitch response (HTR) in mice is considered a behavioral model for hallucinogens and serotonin 5-HT_2A_ receptor function, as well as Tourette syndrome in humans. It is mediated by 5-HT_2A_ receptor agonists such as ( ±)− 2,5-dimethoxy-4-iodoamphetamine (DOI) in the prefrontal cortex (PFC). The 5-HT_2A_ antagonist EMD 281014, can prevent both DOI-induced HTR during ageing and *c-fos* expression in different regions of PFC. Moreover, the nonselective monoamine releaser methamphetamine (MA) suppressed DOI-induced HTR through ageing via concomitant activation of inhibitory 5-HT_1A_ receptors, but enhanced DOI-evoked *c-fos* expression. *d*-Fenfluramine is a selective 5-HT releaser and induces HTR in mice, whereas MA does not. Currently, we investigated whether EMD 281014 or MA would alter: (1) *d*-fenfluramine-induced HTR frequency in 20-, 30- and 60-day old mice, (2) *d*-fenfluramine-evoked c-*fos* expression in PFC, and (3) whether blockade of inhibitory serotonergic 5-HT_1A_- or adrenergic ɑ_2_-receptors would prevent suppressive effect of MA on *d*-fenfluramine-induced HTR.

**Results:**

EMD 281014 (0.001–0.05 mg/kg) or MA (0.1–5 mg/kg) blocked *d*-fenfluramine-induced HTR dose-dependently during ageing. The 5-HT_1A_ antagonist WAY 100635 countered the inhibitory effect of MA on *d*-fenfluramine-induced HTR in 30-day old mice, whereas the adrenergic ɑ_2_ antagonist RS 79948 reversed MA’s inhibitory effect in both 20- and 30- day old mice. *d*-Fenfluramine significantly increased c-*fos* expressions in PFC regions. MA (1 mg/kg) pretreatment significantly increased *d*-fenfluramine-evoked c-*fos* expression in different regions of PFC. EMD 281014 (0.05 mg/kg) failed to prevent *d*-fenfluramine-induced c-*fos* expression, but significantly increased it in one PFC region (PrL at − 2.68 mm).

**Conclusion:**

EMD 281014 suppressed *d*-fenfluramine-induced HTR but failed to prevent *d*-fenfluramine-evoked c-*fos* expression which suggest involvement of additional serotonergic receptors in the mediation of evoked c-*fos*. The suppressive effect of MA on *d*-fenfluramine-evoked HTR is due to well-recognized functional interactions between stimulatory 5-HT_2A_- and the inhibitory 5-HT_1A_- and ɑ_2_-receptors. MA-evoked increases in c-*fos* expression in PFC regions are due to the activation of diverse monoaminergic receptors through increased synaptic concentrations of 5-HT, NE and/or DA, which may also account for the additive effect of MA on *d*-fenfluramine-evoked changes in c-*fos* expression. Our findings suggest potential drug receptor functional interaction during development when used in combination.

**Supplementary Information:**

The online version contains supplementary material available at 10.1186/s12868-022-00766-0.

## Introduction

The head-twitch response (HTR) in mice has been considered as a rodent behavioral model for the: (i) study of serotonergic 5-HT_2A_ receptor function [1], (ii) experimental assessment of hallucinogenic activity observed in humans [1, 2], and iii) investigation of motor symptoms observed in Tourette syndrome (TS) patients [3, 4]. The HTR can be evoked by: (i) serotonin (5-HT) precursors such as 5-hydroxytryptophan [5, 6], ii) direct-acting 5-HT_2A/C_ receptor agonists [e.g., ( ±)− 2,5-dimethoxy-4-iodoamphetamine (DOI) [7], or (iii) selective serotonin releasers, e.g., *d*-fenfluramine [8]. The fact that mice lacking the 5-HT_2A_ receptor cannot exhibit the HTR behavior in response to DOI administration, and restoration of 5-HT_2A_ receptors to cortical neurons in 5-HT_2A_ receptor knockout mice can reinstate the ability of DOI to induce HTR strongly validates the role of 5-HT_2A_ receptors in the induction of HTR [9]. In fact, the central site of initiation of HTR is thought to be within the prefrontal cortex (PFC) since systemic or intra-medial prefrontal cortex (mPFC) injection of DOI produces the HTR in rodents [10–12]. In addition, administration of a variety of nonselective to very selective 5-HT_2A_ receptor antagonists block the DOI-evoked HTR [1, 11, 13]. Taken together, these findings strongly demonstrate that the HTR is a well-studied behavior and its initiation by serotonergic drugs is mediated via activation of postsynaptic 5-HT_2A_ receptors in the PFC.

*d*-Fenfluramine was used clinically as an anorectic agent in the USA [14] but is currently approved for the treatment of epileptic seizures such as Dravet syndrome [15]. It is an amphetamine derivative, whose primary neurochemical effect is thought to be more selective release of 5-HT from serotonergic nerve terminals through its action on serotonin transporter (SERT) [16–19]. *d*-Fenfluramine can evoke HTR behavior in a dose-dependent manner in mice [8]. The efficacy of direct-acting 5-HT_2A_ receptor agonists such as DOI evoking the HTR behavior probably reflects its affinity for 5-HT_2A_ receptors and the efficiency with which DOI-5-HT_2A_-receptor interaction is coupled with its downstream signals [1, 20, 21]. On the other hand, *d*-fenfluramine’s ability in producing the HTR reflects the latter discussed mechanisms, as well as presynaptic factors such as the availability of basal *d*-fenfluramine-sensitive releasable-pool of 5-HT, and the efficiency of 5-HT uptake carrier working in reverse to release serotonin from serotonergic terminals [22–24].

Among synthetic psychoactive substances, methamphetamine (MA) is an amphetamine derivative known as “ice” or “speed” with significant neurotoxic effects and abuse potential [25]. MA is clinically used for the treatment of attention hyperactivity disorder and obesity [26]. MA is classified as a nonselective releaser of monoamines dopamine (DA), norepinephrine (NE), and 5-HT [27, 28], and it is thought to substitute for ligands at the dopamine transporter (DAT), noradrenaline transporter (NET), SERT, and the vesicular monoamine transporter-2 (VMAT-2), which subsequently redistributes monoamines from corresponding storage vesicles into the cytosol by reversing the endogenous function of monoamine transporters. This process results in the release of DA, NE, and 5-HT into corresponding terminal synapses, which respectively activates diverse dopaminergic-, adrenergic-, and serotonergic- receptors [27–29]. MA can also serve as a substrate for trace-amine associated receptor 1 [30] and has affinity for both sigma 1 and sigma 2 receptors [31].

Oftentimes administration of large doses of MA (10–40 mg/kg) in animals under acute or subacute regimens have been utilized to demonstrate consistent evidence for MA-induced long-term physiological and psychological effects as well as CNS pathology including damage to serotonergic and dopaminergic terminals [32–35]. Moreover, more sensitive behavioral models show that lower doses of MA (e.g., 4 × 5 mg/kg at 2 h intervals) in 1 day can increase both the frequency of DOI-induced HTR and corresponding expression of behavioral markers of neuronal activity (c-*fos* and Egr-2) in the mPFC of mice [36]. However, while a single low dose of MA (5 mg/kg) cannot produce the HTR, it does: (1) suppress the DOI-induced HTR in a dose-dependent fashion (1 – 5 mg/kg) in 20-, 30- and 60-day old mice [13], and (ii) increase c-*fos* expression in several regions of the PFC in mice [13].

Age dependency is an important factor in the development of impulsive and suicidal behaviors in children and adolescents with major depressive disorder following their initial treatment with selective serotonin reuptake inhibitor (SSRI) antidepressants [37]. TS is also a commonly diagnosed neurological pediatric disorder which features multiple short and stereotypical motor and phonic tics lasting for one year. The average age of TS onset is 6 years and one half to two thirds of the affected children are virtually symptom before age 18 [38]. The combination of a 5-HT_1A_ antagonist with an SSRI can reverse the observed acute anxiogenic effects of the SSRI when administered in rodents [37]. Clinical management of TS includes aripiprazole, a serotonin 5-HT_1A_- and dopamine D_2_-receptor partial agonist with 5-HT_2A_ receptor antagonist properties [39]. The inhibitory effects of 5-HT_1A_ receptor stimulation on DOI-evoked HTR in mice was recognized in early 1990s [7] and subsequently we demonstrated that blockade of 5-HT_1A_ receptors can prevent the inhibitory effect of the nonselective monoamine reuptake inhibitor cocaine on DOI-evoked HTR [40]. Additionally, selective (e.g., sertraline) and nonselective (e.g., cocaine) serotonin reuptake blockers can attenuate *d*-fenfluramine-evoked HTR in mice via a similar inhibitory mechanism [8]. However, currently there is no published study to demonstrate the ontogenic development of the suppressive effect of the inhibitory serotonin 5-HT_1A_ receptor on the stimulatory 5-HT_2A_ receptor function in the context of a serotonin releaser. Indeed, although MA ranks second highest as the most abused illicit drug in the world [41], neurobiological research concerning the impact of early MA experience on the brain to the development of early-stage addiction is limited.

The developmental ontogeny of HTR behavior following administration of different doses of DOI begins between 14 and 18 days of age, reaches a maximum at day 28, and decreases post 60 days of birth [42]. To date the ontogenic development of *d*-fenfluramine-induced HTR in mice as well as whether acute pretreatment with low doses of MA can suppress the frequency of *d*-fenfluramine-evoked HTR during ageing remain unknown. Thus, based upon our recent study investigating the mechanisms by which MA affects DOI-induced HTR [13], the purpose of the present study was to determine the ontogeny of *d*-fenfluramine-evoked HTR and to investigate whether pretreatment with either MA (0.1–5 mg/kg, i.p.) or the 5-HT_2A_ receptor selective antagonist EMD 281014 (0.001 – 0.05 mg/kg, i.p.; positive control) can alter: (1) the mean frequency of *d*-fenfluramine-induced HTR at different ages (20-, 30- and 60-day old); (2) the expression of c-*fos* evoked by *d*-fenfluramine in different regions of the PFC; and (3) we also investigated whether blockade of the inhibitory serotonergic 5-HT_1A_- [12, 13] or adrenergic ɑ_2_-receptors [13, 43] with their corresponding selective antagonists would alter the suppressive effect of MA on *d*-fenfluramine-induced HTR during development.

## Materials and methods

### Animals and drugs

Albino ICR mice were bred in our animal facility at the Western University of Health Sciences. Male Mice at ages of 20-, 30- and 60-days old were used in this study [13]. Mice were kept in groups of 4–5 on a 12 h light/dark cycle (light 6 am to 6 pm) at a room temperature of 21 ± 2 ℃, with food and water ad libitum. This study was conducted with strict adherence to the recommendations in the guide for the Care and Use of Laboratory Animals of the National Institute of Health (Department of Health and Human Services Publication, revised, 2011). The protocol (#20IACUC013) was approved by the Western University of Health Sciences Institutional Animal Care and Use Committee (IACUC). All efforts were made to both reduce the number of animals used and to minimize their suffering. All experiments were conducted between 9:00 am and 4:00 pm.

*d*-Fenfluramine, the selective 5-HT_2A_ receptor antagonist EMD 281014 (7-{4-[2-(4-fluorophenyl)-ethyl]-piperazine-1-carbonyl}-1H-indole-3-carbonitrile HCl), and the selective ɑ_2_-adrenergic receptor antagonist RS 79948 (RS 79948 hydrochloride) were obtained from Tocris Bioscience (Ellisville, MO, USA). The 5-HT_1A_ receptor selective antagonist WAY 100635 (WAY 100635 maleate salt) and methamphetamine (MA) were purchased from Sigma-Aldrich (St. Louis, MO, USA). All drugs were prepared on the day of the experiment. MA, *d*-fenfluramine, WAY 100635, and RS 79948 were dissolved in distilled water. EMD 281014 was dissolved in 0.2% tween 80 in water [44]. All drugs were administered via i.p. injection at a volume of 0.1 ml/10 g of body weight.

### Behavioral experiments

The HTR is a very distinctive head-twitching behavior in mice and usually cannot be mistaken for such behaviors as head shakes (lateral movement of the head from side to side) or head jerks (up and down jerking) [45]. Each mouse was individually transferred to a plastic cage (40 X 25 X 16 cm) and acclimated to the test environment for a period of 30 min prior to the first injection. To evaluate the effect of MA or the selective 5-HT_2A_ receptor antagonist EMD 281014 on the *d*-fenfluramine-induced HTR during different ages, 20-, 30- and 60-day old mice [13] were randomly divided into different groups and were pretreated with either the corresponding vehicle (i.p.), or varying doses of MA (0.1 – 5 mg/kg, i.p.) or EMD 281014 (0.001 – 0.05 mg/kg, i.p.) at time 0. Each mouse was then injected with a dose of *d*-fenfluramine (5 mg/kg, i.p.) [8, 22] at 30 min. Each mouse was individually observed immediately following the injection of *d*-fenfluramine and the HTR score (mean ± SEM) was recorded cumulatively at 5-min intervals for the next 30 min. To investigate the ability of the selective 5-HT_1A_ receptor antagonist WAY 100635 or the selective ɑ_2_-adrenergic receptor antagonist RS 79948 to reverse the inhibitory effect of MA on *d*-fenfluramine-induce HTR, we performed these studies in 20- and 30-day old mice since 60-day old animals produced few HTRs in response to *d*-fenfluramine. Thus, mice were separated into groups, and were injected with either the corresponding vehicle (i.p.), or a single dose of MA (1 mg/kg, i.p.) at 0 min [13]. The treated mice were then treated with either WAY 100635 (0.25 mg/kg, i.p.) or RS 79948 (0.1 mg/kg, i.p.) at 20 min [13]. Ten minutes later, these mice received an injection of *d*-fenfluramine (5 mg/kg, i.p.). The *d*-fenfluramine evoked HTR (mean ± SEM) was then recorded as described above. The observers were blind to animals’ treatment conditions.

### c-fos immunohistochemistry and image analysis

To investigate the specific activation of sub-regions of PFC induced by the injection of *d*-fenfluramine (5 mg/kg, i.p.), and whether pretreatment with either MA (1 mg/kg, i.p.) or EMD 281014 (0.05 mg/kg, i.p.) could alter the expression of c-*fos* evoked by *d*-fenfluramine in a differential manner across different regions in the PFC, 30 days old mice were selected to perform the c-*fos* immunohistochemistry study because at this age mice produced a maximal HTR frequency in response to *d*-fenfluramine administration.

Two hours after the first injection [13], mice were deeply anesthetized with isoflurane (3%) and were then transcardially perfused with 0.01 M phosphate buffered saline (PBS) followed by 4% paraformaldehyde. The brains were removed immediately and put in the same fixative solution for 2 h, and then placed in 0.1 M PB containing 30% sucrose until they sank. The coronal sections of the PFC were cut at 25 µm using a cryostat (Leica, Bannockburn, IL, USA), and pre-incubated in the blocking buffer (0.01 M PBS containing 10% normal donkey serum and 0.3% Triton X-100) for 1 h at room temperature. Then the slices were incubated in a rabbit anti-c-*fos* polyclonal antibody (1:1000; Abcam) in 0.01 M PBS containing 5% normal donkey serum, 0.05% sodium azide, and 0.3% Triton X-100 at 4 ℃ overnight. After washing in PBS for 3 times, the slices were then incubated in an Alexa 594-conjugated goat anti-rabbit secondary antibody (1:1000; Invitrogen) in 0.01 M PBS containing 0.3% Triton X-100 for 4 h at room temperature. The sections were washed for 3 times, then were mounted and coverslipped with an anti-fade mounting medium containing DAPI (Vector Laboratories).

Images were captured by a confocal laser-scanning microscope (Zeiss LSM 880) with Zen software utilizing tile-scan imaging at 20X magnification. Magnified images were further acquired at 60X magnification and 1024 × 1024 pixels. Images for all groups in each experiment were obtained using identical acquisition parameters and analyzed using ImageJ software (NIH). As described previously [13], the c-*fos* numbers of the sub-regions of three consecutive sections at the 5 following coronal levels of mice PFC were analyzed, bregma − 2.68 mm [frontal associated cortex (FrA), prelimbic cortex (PrL), medial orbital cortex (MO), ventral orbital cortex (VO), lateral orbital cortex (LO), dorsal lateral orbital cortex (DLO)], bregma − 2.34 and −  2.1 mm [primary motor cortex (M1), secondary motor cortex (M2), cingulate cortex area 1 (Cg1), PrL, MO, VO, LO, agranular insular cortex (AI)], bregma − 1.98 mm [primary somatosensory area (S1), M1, M2, Cg1, PrL, infralimbic cortex (IL), MO, VO, LO, AI], and bregma − 1.7 mm [S1, M1, M2, Cg1, PrL, IL, dorsal peduncular cortex (DP)] [46]. c-*fos* positive nuclei of each region were counted when the cell nucleus was round or oval, filled and double-labeled with DAPI. The mean value of the c-*fos* number in each area was calculated from three consecutive sections of an individual mouse brain and was used in statistical analysis. Acquisition and analysis of images were carried out under blind experimental condition.

### Statistical analysis

Statistical analyses were done using Graphpad Prism 8 (Graphpad software Inc., San Diego, CA). The frequency of HTR induced by *d*-fenfluramine in 20-, 30-, or 60-day old mice, as well as histological data were analyzed by one-way analysis of variance (ANOVA) followed by Tukey's multiple test. The effect of varying doses of MA on the *d*-fenfluramine-induced HTR in 20-, 30-, and 60-day old mice were analyzed using the Kruskal–Wallis non-parametric one-way ANOVA followed by Dunn’s post hoc test. The effect of EMD 281014 (0.001– 0.05 mg/kg, i.p.) on the *d*-fenfluramine-induced HTR in 20-, 30-, and 60-day old mice, and the effect of WAY 100,635 (0.25 mg/kg, i.p.) / RS 79,948 (0.1 mg/kg, i.p.) on the inhibitory effect of MA (1 mg/kg, i.p.) on *d*-fenfluramine-induced HTR in 20- and 30-day old mice were analyzed by two-way ANOVA followed by Dunnett’s test for multiple comparisons. All data were expressed as mean ± SEM. A *p* value of less than 0.05 was considered significant.

## Results

### d-Fenfluramine (5 mg/kg, i.p.) evoked a maximal frequency of HTRs in 30-day old mice

**Fig. 1:**
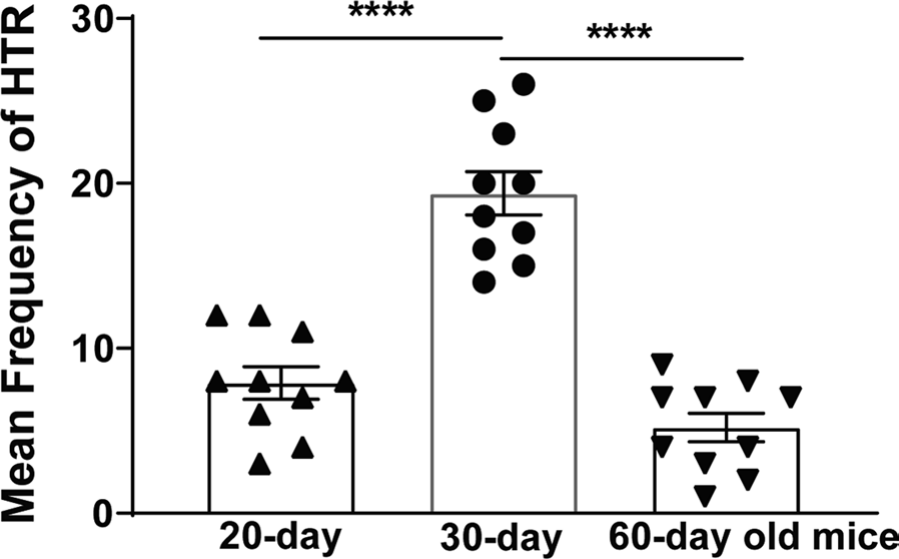
The individual as well as mean (± SEM) frequency of HTR induced by *d*-fenfluramine (5 mg/kg, i.p.) across different ages (20-, 30- and 60-day old) in mice. Significant differences occurring between 20- and 30-day (^****^p < 0.0001) and between 30- and 60-day old mice (^****^p < 0.0001). One-way ANOVA followed by Tukey's test. *n* = 10 in each age group. Data are presented as means ± SEM

 d-Fenfluramine (5 mg/kg, i.p.) [8, 22] produced HTRs in mice in a bell-shaped manner when tested across ages 20-, 30- and 60-days (Fig. 1). One-way ANOVA showed significant differences in the mean frequency of HTR among the ages (*F *_*2, 27*_ = 49.38, *p* < 0.0001; Fig. 1). Compared to the 20-day old mice, the mean frequency of *d*-fenfluramine-induced HTR significantly increased by 146% in 30-day old mice (*p* < 0.0001, *n* = 10 mice per age group, Tukey's test), which subsided by 73% in 60-day old mice (*p* < 0.0001, *n* = 10 mice per age group; Tukey's test). These findings indicate that administration of a 5 mg/kg dose of *d*-fenfluramine produces a maximal mean frequency of HTR during postnatal day 30.

### The selective 5-HT_2A_ receptor antagonist EMD 281014 dose-dependently inhibits *d*-fenfluramine-induced HTR across the age-range tested

**Fig. 2:**
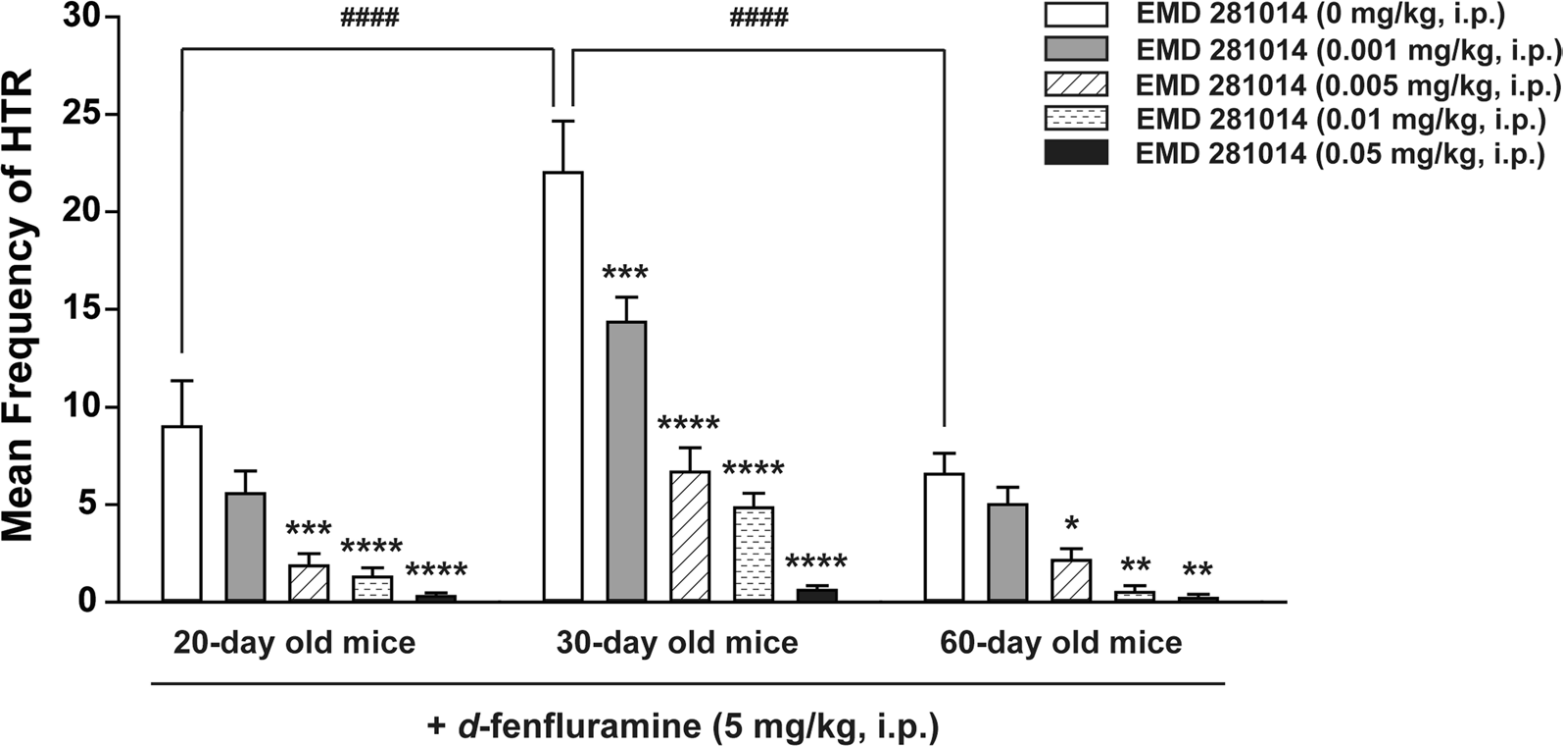
Suppressive effects of varying doses of the selective 5-HT_2A_ receptor antagonist EMD 281014 (0, 0.001, 0.005, 0.01 and 0.05 mg/kg, i.p.) on the mean (± SEM) frequency of HTR induced by *d*-fenfluramine (5 mg/kg, i.p.) in 20-, 30- and 60-day old mice. Among the EMD 281014 vehicle-pretreated control age groups, significant differences in the mean frequency of *d*-fenfluramine-induced HTR were observed between 20- and 30-day (^####^*p* < 0.0001) and between 30- and 60-day old mice (^####^*p* < 0.0001). Varying doses of EMD 281014 suppressed *d*-fenfluramine-induced HTR in a dose-dependent manner across the age-range tested. Compared to corresponding age-matched vehicle-pretreated control group, significant reductions were observed at all tested doses of EMD 281014 in 30-day old mice, but only at 0.005, 0.01 and 0.05 mg/kg doses in 20- and 60-day old mice. *p < 0.05, **p < 0.01, ***p < 0.001, ****p < 0.0001 *vs*. vehicle injection; two-way ANOVA followed by Dunnett's test. *n* = 5 − 9 in each group. Data are presented as means ± SEM

As in our previous study [13], administration of varying doses of the 5-HT_2A_ receptor antagonist EMD 281014 (0.001, 0.005, 0.01, and 0.05 mg/kg, i.p.) by itself did not affect the basal HTR scores in 20-, 30-, and 60-days old mice (all 0 ± 0; *n* = 6 per group). A two-way ANOVA (age × dose of drug) showed significant effects on the frequency of *d*-fenfluramine-induced HTR for age (*F*_*2, 84*_ = 41.96, *p* < 0.0001), dose of EMD 281014 (*F*_*4, 84*_ = 48.64, *p* < 0.0001), and their interaction (*F*_*8, 84*_ = 5.954, *p* < 0.0001; Fig. 2). Among EMD 281014 vehicle-pretreated control age groups, significant differences in the mean frequency of *d*-fenfluramine-induced HTR were observed between 20- and 30-day (*p* < 0.0001; Dunnett's test) and between 30- and 60-day old mice (*p* < 0.0001; Dunnett's test; Fig. 2). Relative to the corresponding age-matched vehicle-pretreated control group, varying doses of EMD 281014 (0.001, 0.005, 0.01 and 0.05 mg/kg, i.p., *n* = 5 − 9 mice per group) suppressed *d*-fenfluramine-induced HTR in a dose-dependent manner across the age-range tested, with respective ID_50_ values of 0.001367 (0.00082 − 0.002187 mg/kg, in 20-day old mice), 0.001947 (0.001455 − 0.002577 mg/kg, in 30-day old mice), and 0.00214 (0.001219 − 0.003619 mg/kg, in 60-day old mice). In 20- and 60-day old mice, EMD 281014 significantly suppressed the frequency of *d*-fenfluramine-induced HTR at 0.005 (*p* = 0.0002 for 20-day old mice, *p* = 0.0254 for 60-day old mice; Dunnett's test), 0.01 (*p* < 0.0001 for 20-day old mice, *p* = 0.002 for 60-day old mice; Dunnett's test), and 0.05 mg/kg (*p* < 0.0001 for 20-day old mice, *p* = 0.0022 for 60-day old mice; Dunnett's test), whereas significant decreases were observed at all tested doses of EMD 281014 in 30-day old mice (*p* = 0.0003 for 0.001 mg/kg, all other *p* < 0.0001; Dunnett's test; Fig. 2).

### MA dose-dependently suppresses *d*-fenfluramine-induced HTR across the age-range tested

**Fig. 3:**
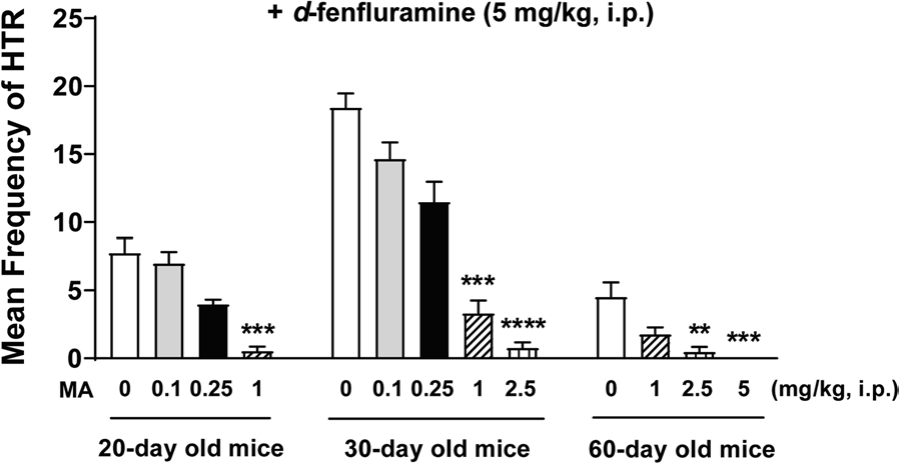
Suppressive effects of varying doses of MA on the mean (± SEM) frequency of HTR induced by *d*-fenfluramine (5 mg/kg, i.p.) in 20 (MA at 0, 0.1, 0.25, 1 mg/kg, i.p.)-, 30 (MA at 0, 0.1, 0.25, 1, 2.5 mg/kg, i.p.)- and 60 (MA at 0, 1, 2.5, 5 mg/kg, i.p.)-day old mice. Varying doses of MA inhibited *d*-fenfluramine-induced HTR in a dose-dependent manner across the age-range tested. Compared to corresponding age-matched vehicle-pretreated control group, significant reductions were observed at 1 mg/kg in 20-day old mice, at 1 and 2.5 mg/kg in 30-day old mice, at 2.5 and 5 mg/kg in 60-day old mice, respectively. **p < 0.01, ***p < 0.001, ****p < 0.0001 *vs*. vehicle injection; Kruskal–Wallis non-parametric one-way ANOVA followed by Dunn’s test. *n* = 5 − 9 in each group. Data are presented as means ± SEM

As in our previous study [13], injection of MA (0.1, 0.25, 1, 2.5, 5 mg/kg, i.p.) by itself had no effect on basal HTR frequency in 20-, 30-, and 60-day old mice (all 0 ± 0; *n* = 6 per group). In the current study, MA dose-dependently suppressed the *d*-fenfluramine-induced HTR in 20 (KW _*4, 27*_ = 18.61, *p* = 0.0003; *n* = 5 − 9 mice per group)-, 30 (KW _*5, 32*_ = 25.64, *p* < 0.0001; *n* = 5 − 9 mice per group) -, and 60 (KW _*4, 25*_ = 18.08, *p* = 0.0004; *n* = 5 − 9 mice per group) -day old mice (Kruskal–Wallis non-parametric one-way ANOVA; Fig. 3). Relative to the corresponding age-matched vehicle-pretreated control group, MA significantly suppressed the frequency of *d*-fenfluramine-induced HTR at 1 mg/kg in 20-day old mice (*p* = 0.0002, Dunn’s test), at 1 (*p* = 0.0007, Dunn’s test) and 2.5 mg/kg (*p* < 0.0001, Dunn’s test) in 30-day mice, and at 2.5 (*p* = 0.0042, Dunn’s test) and 5 mg/kg (*p* = 0.0005, Dunn’s test) in 60-day mice (Fig. 3), with respective ID_50_ values of 0.2407 (0.1431 − 0.4018 mg/kg, in 20-day old mice), 0.2961 (0.2088 − 0.4185 mg/kg, in 30-day old mice), and 0.4811 (0.2342 − 0.8154 mg/kg, in 60-day old mice).

### The impact of blocking inhibitory serotonergic 5-HT_1A_- and adrenergic ɑ_2_- receptors on the suppressive effect of MA on *d*-fenfluramine-induced HTR

Since *d*-fenfluramine could not produce a substantial number of HTRs in 60-day old mice, we carried out the inhibitory receptor interaction studies in 20- and 30-day old mice. We have previously shown that the selective 5-HT_1A_ receptor antagonist WAY 100635 can reverse the inhibitory effect of MA on DOI-induced HTR [13], and likewise the ɑ_2_-adrenergic receptor antagonist yohimbine can prevent the inhibitory effects of the monoamine reuptake blocker cocaine on DOI-induced HTR [40]. In the current study a 1 mg/kg dose of MA significantly decreased the frequency of *d*-fenfluramine (5 mg/kg, i.p.)-induced HTR in 20- and 30-day old mice, and thus we investigated whether pretreatment with the selective 5-HT_1A_ receptor antagonist WAY 100635, or the selective ɑ_2_-adrenergic receptor antagonist RS 79948, would also reverse the suppressive effect of MA on *d*-fenfluramine-induced HTR in 20- and 30-day old mice.

**Fig. 4:**
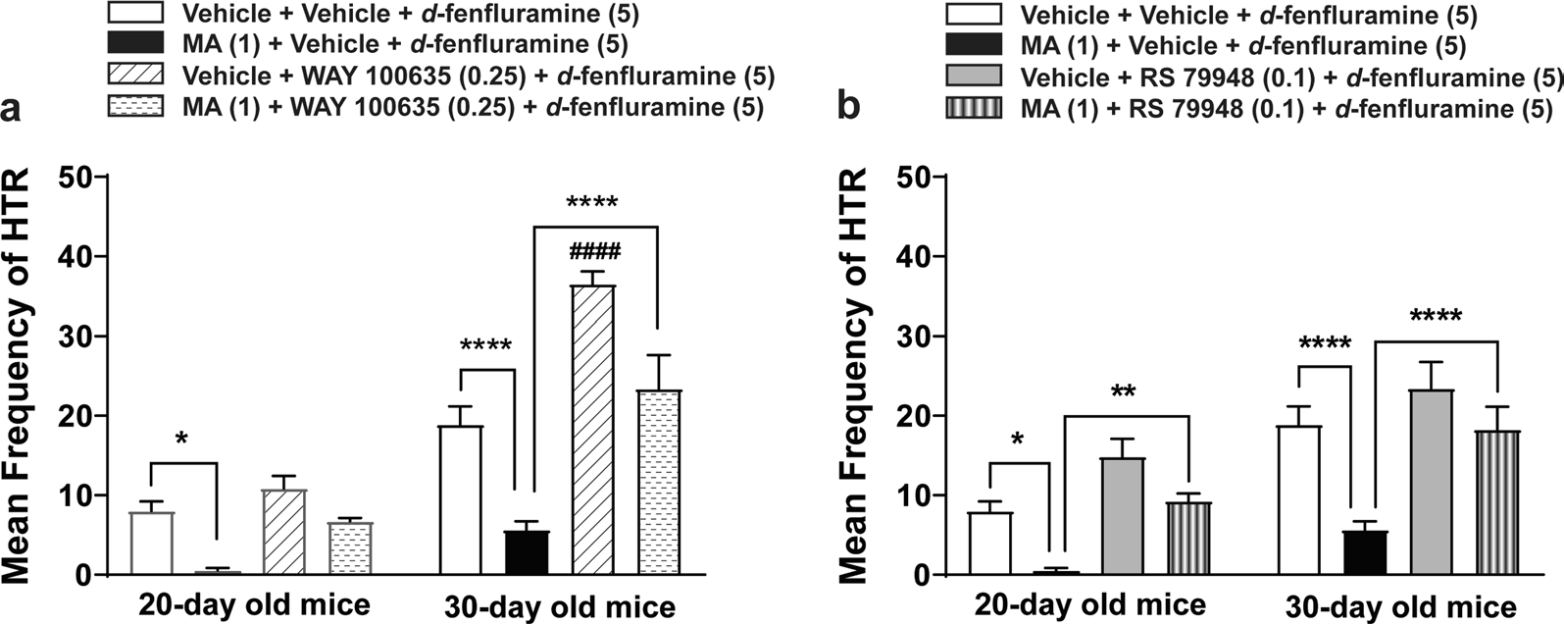
Pretreatment with either the selective 5-HT_1A_ receptor antagonist WAY 100635 (0.25 mg/kg, i.p., a), or the selective ɑ_2_-adrenergic receptor antagonist RS 79948 (0.1 mg/kg, i.p., b), reversed the inhibitory action of MA (1 mg/kg, i.p.) on *d*-fenfluramine-induced (5 mg/kg, i.p.) HTR in 20- and 30-day old mice. Relative to the corresponding age-matched Vehicle + Vehicle + *d*-fenfluramine pretreated group, MA pretreatment (i.e. MA + Vehicle + *d*-fenfluramine treatment group) significantly reduced the frequency of *d*-fenfluramine-induced HTR in 20- (*p = 0.0342) and 30-day old mice (****p < 0.0001; **a**, **b**); whereas WAY 100635 pretreatment (i.e. Vehicle + WAY 100635 + *d*-fenfluramine treatment group) significantly potentiated the frequency of *d*-fenfluramine-induced HTR in 30-day (^####^*p* < 0.0001, a) but not in 20-day old mice. Relative to the corresponding MA + Vehicle + *d*-fenfluramine treatment group, inclusion of WAY 100635 (i.e. the MA + WAY 100635 + *d*-fenfluramine treatment group) only significantly reversed the inhibitory effect of MA on DOI-induced HTR in 30-day old mice (****p < 0.0001, a); whereas inclusion of RS 79948 (i.e. the MA + RS 79948 + *d*-fenfluramine treatment group) significantly reversed the inhibitory effect of MA on DOI-induced HTR in both 20 (**p = 0.0082, b)- and 30 (****p < 0.0001, b)-day old mice. Two-way ANOVA followed by Dunnett's test. *n* = 6 − 9 in each group. Data are presented as means ± SEM

For the WAY 100635 experiment, a two-way ANOVA (age × treatment) showed highly significant differences among the ages (*F*_*1, 52*_ = 95.91, *p* < 0.0001), treatment (*F*_*3, 52*_ = 30.49, *p* < 0.0001), and age × treatment interaction (*F*_*3, 52*_ = 8.202, *p* = 0.0001; Fig. 4a). Indeed, relative to the corresponding age-matched vehicle-pretreated control group (i.e. vehicle + vehicle + *d*-fenfluramine, *n* = 8 − 9 mice per age group), MA (1 mg/kg, i.p.) pretreatment (i.e. the MA + Vehicle + *d*-fenfluramine group, *n* = 7 − 8 mice per age group) significantly decreased the frequency of *d*-fenfluramine-induced HTR in 20- and 30-day mice (*p* = 0.0342 for 20- day old mice, *p* < 0.0001 for 30-day mice; Dunnett's test; Fig. 4a). Inclusion of the 5-HT_1A_ antagonist WAY 100635 (0.25 mg/kg, i.p.) (i.e. the MA + WAY 100635 + *d*-fenfluramine treatment group, *n* = 7 − 8 mice per age group) reversed the inhibitory effect of MA on *d*-fenfluramine-induced HTR, but significance was observed only in 30-day old mice (*p* < 0.0001; Dunnett’s test; Fig. 4a). In addition, WAY 100635 (0.25 mg/kg, i.p.) by itself significantly potentiated the frequency of *d*-fenfluramine-induced HTR in 30-day old mice (*p* < 0.0001, *n* = 6; Dunnett’s test; Fig. 4a).

For the RS 79948 experiment, a two-way ANOVA (age × treatment) showed highly significant differences among the ages (*F*_*1, 52*_ = 36.11, *p* < 0.0001), treatment (*F*_*3, 52*_ = 22.19, *p* < 0.0001), but not for age × treatment interaction (*F*_*3, 52*_ = 0.801, *p* = 0.499; Fig. 4b). Inclusion of RS 79948 (0.1 mg/kg, i.p.) (i.e., the MA + RS 79948 + *d*-fenfluramine treatment group, *n* = 7 − 8 mice per age group) significantly reversed the inhibitory effect of MA on *d*-fenfluramine-induced HTR in 20 (*p* = 0.0082; Dunnett's test)- and 30 (*p* < 0.0001; Dunnett's test; Fig. 4b)-day old mice. RS 79948 (0.1 mg/kg, i.p.) by itself did not significantly enhance the frequency of *d*-fenfluramine-induced HTR in both 20- and 30-day old mice.

### The selective 5-HT_2A_ receptor antagonist EMD 281014 did not prevent *d*-fenfluramine-induced c-*fos* expression in the mouse PFC

**Fig. 5:**
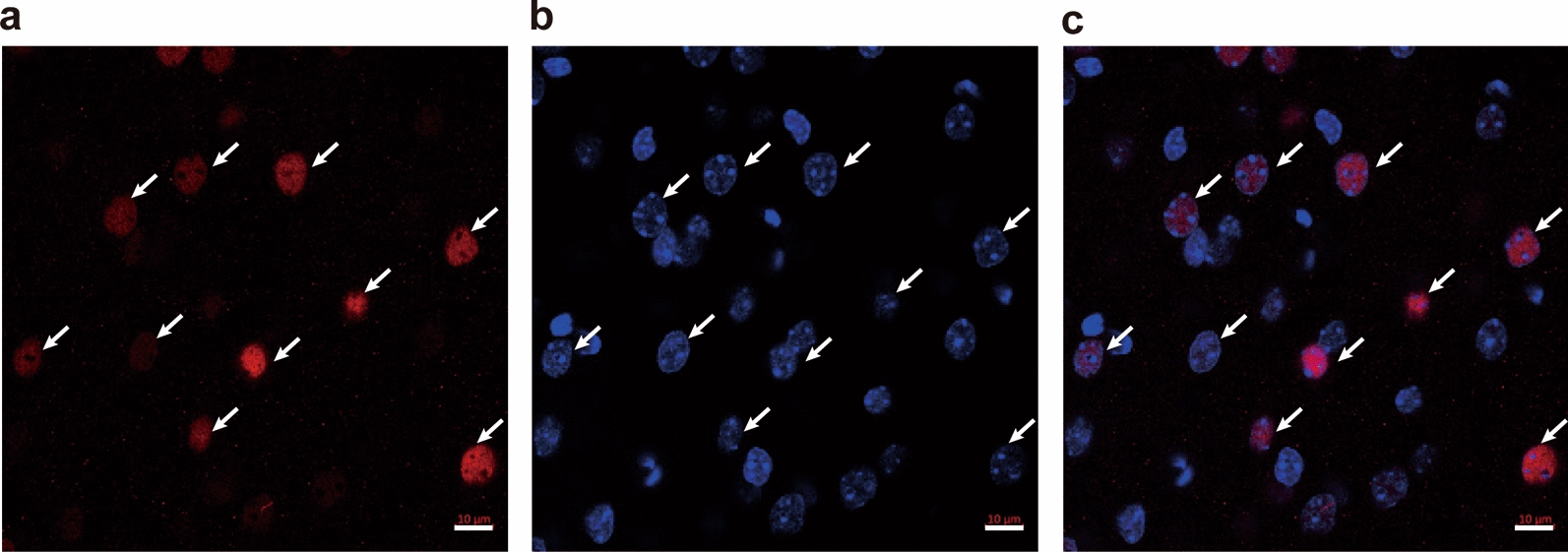
Double-labeled immunofluorescent staining for c-*fos* (red; **a**), DAPI (blue; **b**) and the merged image (**c**) in mice PFC. c-*fos* immunoreactivity was counted when the cell nucleus was round or oval, completely filled, and double-labeled with DAPI, scale bars = 10 μm

**Fig. 6:**
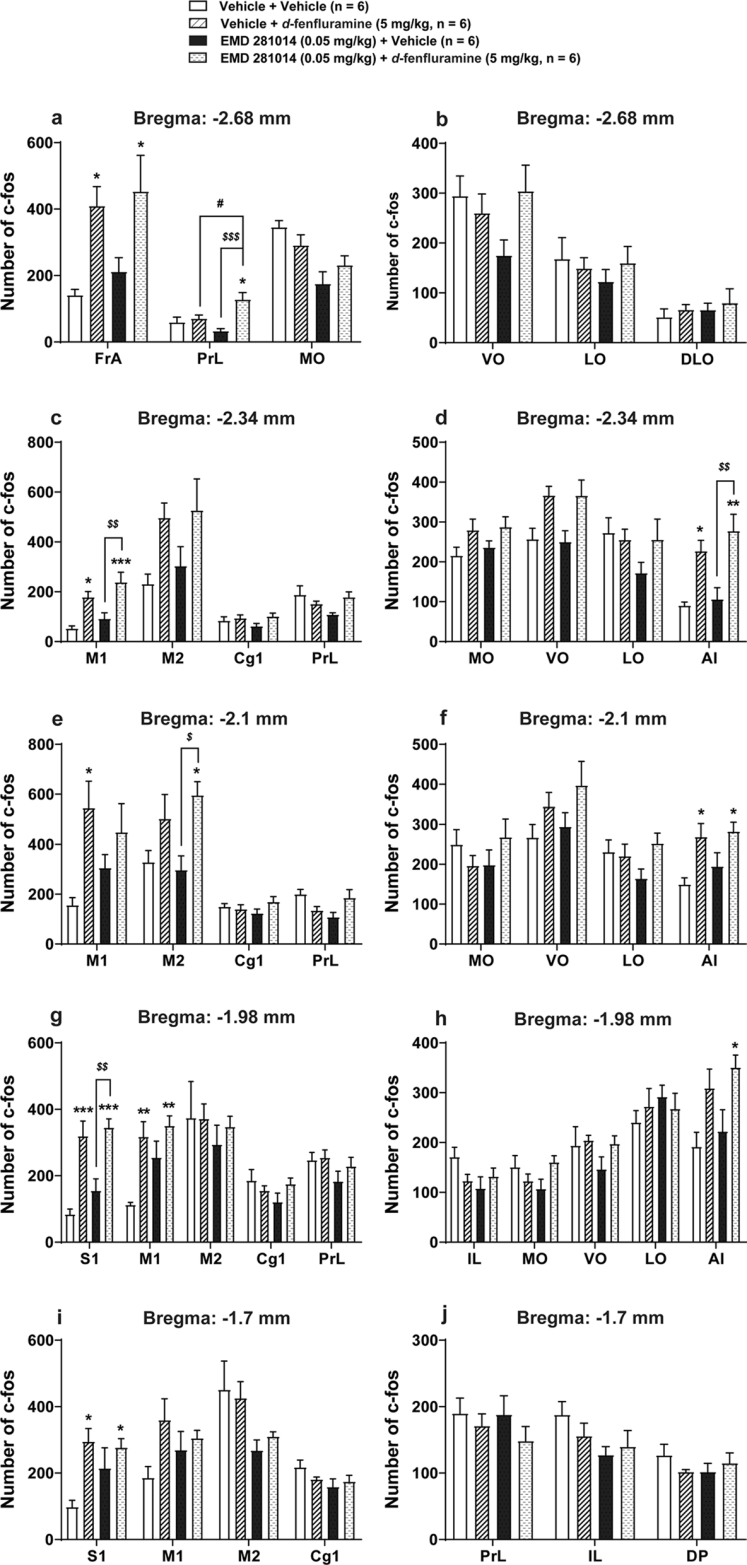
Effects of the selective 5-HT_2A_ receptor antagonist EMD 281014 on *d*-fenfluramine-induced c-*fos* expression in different regions at 5 coronal sections in the PFC of mice. Compared to the Vehicle + Vehicle control group, administration of *d*-fenfluramine (i.e. Vehicle + *d*-fenfluramine group) significantly induced c-*fos* expressions in the: (1) FrA at the level of − 2.68 mm relative to bregma (**a**); (2) M1 and AI at the level of − 2.34, and − 2.1 mm relative to bregma (**c**–**f**); (3) S1 and M1 at the level of − 1.98 mm relative to bregma (**g**); (4) S1 at the level of − 1.7 mm relative to bregma (**i**). Compared to the Vehicle + Vehicle group, EMD 281014 by itself (i.e. EMD 281014 + Vehicle group) did not cause any changes in c-*fos* expression in the PFC, whereas inclusion of *d*-fenfluramine (i.e. EMD 281014 + *d*-fenfluramine treatment group) significantly increased c-*fos* expression in the: (1) FrA and PrL at the level of − 2.68 mm relative to bregma (**a**); (2) M1 and AI at the level of − 2.34 mm relative to bregma (**c**, **d**); (3) M2 and AI at the level of − 2.1 mm relative to bregma (**e**, **f**); (4) S1, M1 and AI at the level of − 1.98 mm relative to bregma (**g**, **h**); (5) S1 at the level of − 1.7 mm relative to bregma (**i**). Compared to the EMD 281014 + Vehicle treatment group, inclusion of *d*-fenfluramine (i.e. EMD 281014 + *d*-fenfluramine treatment group) significantly increased c-*fos* expression in the: (1) PrL at the level of − 2.68 mm relative to bregma (**a**); (2) M1 and AI at the level of − 2.34 mm relative to bregma (**c**, **d**); (3) M2 at the level of − 2.1 mm relative to bregma (**e**); (4) S1 at the level of − 1.98 mm relative to bregma (**g**). However, EMD 281014 did not prevent *d*-fenfluramine -induced c-*fos* expressions in the areas of the PFC (i.e., EMD 281014 + *d*-fenfluramine treatment group vs. Vehicle + *d*-fenfluramine treatment group, (**a**–**j**). In addition, pre-treatment with EMD 281014 significantly increased *d*-fenfluramine-induced c-*fos* expression in the PrL at the level of − 2.68 mm relative to bregma (a). *p < 0.05, **p < 0.01, ***p < 0.001 *vs*. Vehicle + Vehicle pretreated control-mice; ^#^*p* < 0.05 *vs.* Vehicle + *d*-fenfluramine treatment group; ^*$*^*p* < 0.05, ^*$$*^*p* < 0.01, ^*$$$*^*p* < 0.001 *vs*. EMD 281014 + Vehicle group; one-way ANOVA followed by Tukey's test. Data are presented as means ± SEM

Figure 5 shows *d*-fenfluramine-induced c-*fos* expression double-labeled with DAPI in the PFC. Following our previous protocols [13], in the current study we investigated the number of c*-fos* expression induced by *d*-fenfluramine in different regions of PFC sections of each mouse brain under various experimental conditions at 5 coronal levels [46]. In mice treated with vehicle + vehicle (*n* = 6), mild basal levels of c-*fos* immunoreactivity were detectd in different areas of the PFC (Fig. 6). Compared to the corresponding vehicle-treated sections, administration of *d*-fenfluramine (i.e. Vehicle + *d*-fenfluramine (5 mg/kg, i.p.), *n* = 6) induced significant c-*fos* expression in the: 1) FrA (*p* = 0.0434) at the level of − 2.68 mm relative to bregma (Fig. 6a); 2) M1 (*p* = 0.0142) and AI (*p* = 0.017) at the level of − 2.34 mm relative to bregma (Fig. 6c, d); 3) M1 (*p* = 0.0183) and AI (*p* = 0.0328) at the level of − 2.1 mm relative to bregma (Fig. 6e, f); 4) S1 (*p* = 0.0003) and M1 (*p* = 0.0046) at the level of − 1.98 mm relative to bregma (Fig. 6 g); 5) S1 (*p* = 0.0126) at the level of − 1.7 mm relative to bregma (Fig. 6i). Compared to the control group, the selective 5-HT_2A_ receptor antagonist EMD 281014 by itself (i.e. EMD 281014 (0.05 mg/kg, i.p.) + vehicle group, *n* = 6) did not cause any changes in c-*fos* expression in the PFC, whereas inclusion of *d*-fenfluramine (i.e. EMD 281014 (0.05 mg/kg, i.p.) + *d*-fenfluramine (5 mg/kg, i.p.) treatment group) significantly increased c-*fos* expression in the: 1) FrA (*p* = 0.0162) and PrL (*p* = 0.0158) at the level of – 2.68 mm relative to bregma (Fig. 6a); 2) M1 (*p* = 0.0004) and AI (*p* = 0.0011) at the level of − 2.34 mm relative to bregma (Fig. 6c, d); 3) M2 (*p* = 0.0456) and AI (*p* = 0.0156) at the level of − 2.1 mm relative to bregma (Fig. 6e, f); 4) S1 (*p* = 0.0001), M1 (*p* = 0.0011) and AI (*p* = 0.0209) at the level of − 1.98 mm relative to bregma (Fig. 6g, h); 5) S1 (*p* = 0.025) at the level of − 1.7 mm relative to bregma (Fig. 6i). Compared to the EMD 281014 alone treatment (i.e. EMD 281014 (0.05 mg/kg, i.p.) + vehicle treatment group), inclusion of *d*-fenfluramine (i.e. EMD 281014 (0.05 mg/kg, i.p.) + *d*-fenfluramine (5 mg/kg, i.p.) treatment group) significantly increased c-*fos* expression in the: 1) PrL (*p* = 0.0009) at the level of − 2.68 mm relative to bregma (Fig. 6a); 2) M1 (*p* = 0.0041) and AI (*p* = 0.0026) at the level of − 2.34 mm relative to bregma (Fig. 6c, d); 3) M2 (*p* = 0.0229) at the level of − 2.1 mm relative to bregma (Fig. 6e); 4) S1 (*p* = 0.0031) at the level of − 1.98 mm relative to bregma (Fig. 6g). However, EMD 281014 did not prevent *d*-fenfluramine-induced c-*fos* expressions in the areas of the PFC examined (i.e. EMD 281014 (0.05 mg/kg, i.p.) + *d*-fenfluramine (5 mg/kg, i.p.) treatment group vs. Vehicle + *d*-fenfluramine (5 mg/kg, i.p.) treatment group, Fig. 6a–j). Unexpectedly, it significantly increased c-*fos* expression in the PrL region (*p* = 0.0494) at the level of − .68 mm relative to bregma (Fig. 6a).

### MA significantly increased *d*-fenfluramine-induced c-*fos* expression in mice PFC

**Fig. 7:**
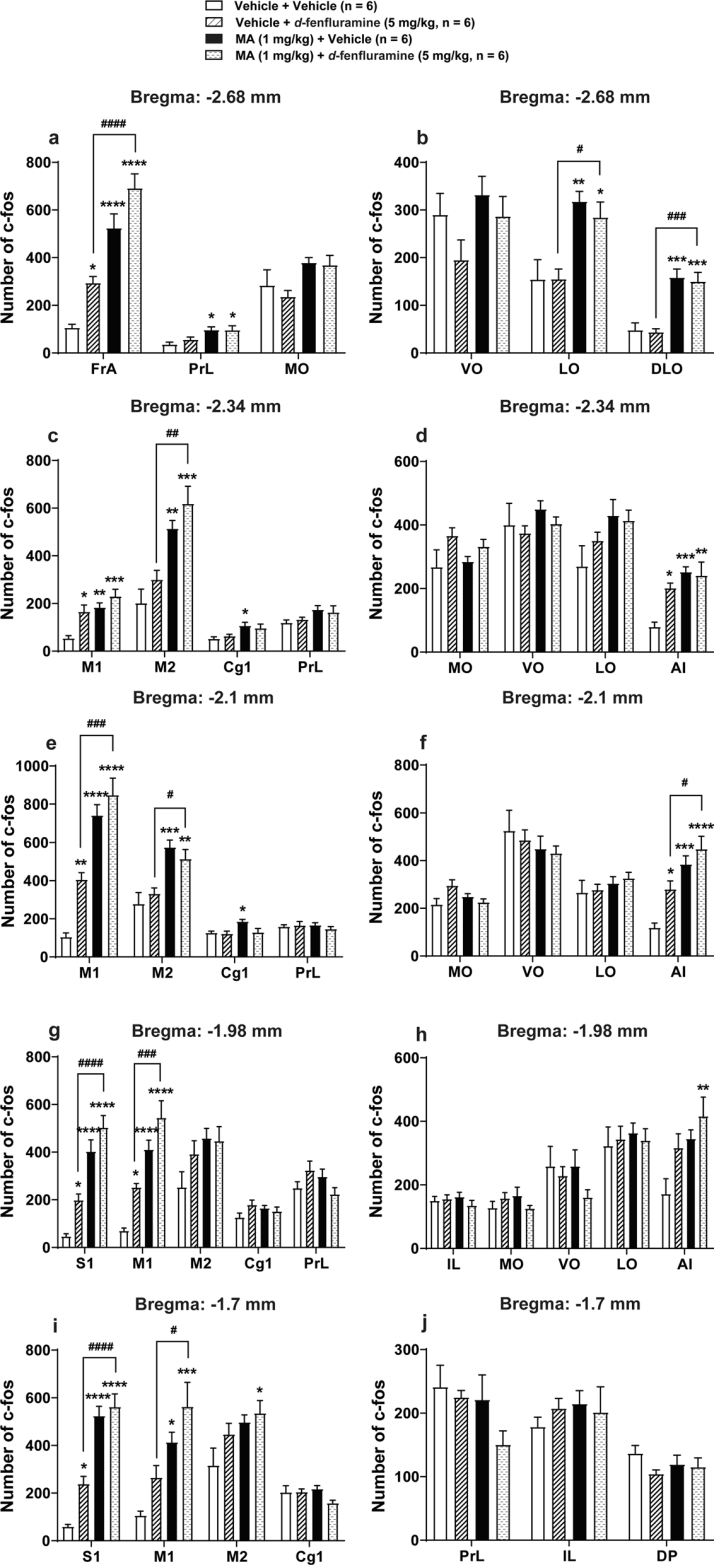
Effects of MA on *d*-fenfluramine-induced c-*fos* expression in different regions at 5 coronal sections in the PFC of mice. Relative to Vehicle + Vehicle group, *d*-fenfluramine by itself (i.e., Vehicle + *d*-fenfluramine) significantly increased c-*fos* expression in the areas in mice PFC as described in Fig. 6. Compared to the Vehicle + Vehicle control group, MA by itself (i.e. MA + Vehicle group) significantly increased c-*fos* expressions in the: (1) FrA, PrL, LO and DLO at the level of − 2.68 mm relative to bregma (**a**, **b**); (2) M1, M2, Cg1 and AI at the level of − 2.34 and − 2.1 mm relative to bregma (**c**–**f**); (3) S1 and M1 at the level of − 1.98 and − 1.7 mm relative to bregma (g, i); inclusion of *d*-fenfluramine (i.e. MA + *d*-fenfluramine treatment group) significantly increased c-*fos* expression in the: (1) FrA, PrL, LO and DLO at the level of − 2.68 mm relative to bregma (**a**, **b**); (2) M1, M2 and AI at the level of − 2.34 and − 2.1 mm relative to bregma (**c**–**f**); (3) S1, M1 and AI at the level of − 1.98 mm relative to bregma (**g**, **h**); (4) S1, M1 and M2 at the level of − 1.7 mm relative to bregma (**i**). Compared to the Vehicle + *d*-fenfluramine group, pre-treatment with MA (i.e. MA + *d*-fenfluramine group) significantly increased c-*fos* expression in the: (1) FrA, LO and DLO at the level of − 2.68 mm relative to bregma (**a**, **b**); (2) M2 at the level of − 2.34 mm relative to bregma (**c**); (3) M1, M2 and AI at the level of − 2.1 mm relative to bregma (**e**, **f**); (4) S1 and M1 at the level of − 1.98 and − 1.7 mm relative to bregma (**g**, **i**). Neither MA alone nor in combination with *d*-fenfluramine produced any significant changes in c-*fos* expression in the: (1) MO, VO at the level of − 2.68 mm relative to bregma (a, b); (2) PrL, MO, VO and LO at the level of − 2.34 and − 2.1 mm relative to bregma (**c**–**f**); (3) M2, Cg1, PrL, IL, MO, VO and LO at the level of − 1.98 mm relative to bregma (**g**, **h**); and (4) Cg1, PrL, IL and DP at the level of − 1.7 mm relative to bregma (**i**, **j**). *p < 0.05, **p < 0.01, ***p < 0.001, ****p < 0.0001 *vs*. Vehicle + Vehicle group; ^*#*^*p* < 0.05, ^*##*^*p* < 0.01, ^*###*^*p* < 0.001, ^*####*^*p* < 0.0001 *vs.* Vehicle + *d*-fenfluramine group; one-way ANOVA followed by Tukey's test. Data are presented as means ± SEM

*d*-Fenfluramine (5 mg/kg, i.p.) also evoked significant increases in c-*fos* expressions in the same regions of the PFC in MA-vehicle treated mice (*n* = 6, Fig. 7) as already described in the EMD 281014-vehicle treated mice (Fig. 6). Compared to the vehicle + vehicle group, MA by itself (i.e. MA (1 mg/kg, i.p.) + vehicle group) significantly increased c-*fos* expressions in the: 1) FrA (*p* < 0.0001), PrL (*p* = 0.0265), LO (*p* = 0.0061) and DLO (*p* = 0.0004) at the level of – 2.68 mm relative to bregma (Fig. 7a, b); 2) M1 (*p* = 0.0047), M2 (*p* = 0.0029), Cg1 (*p* = 0.0314) and AI (*p* = 0.0005) at the level of − 2.34 mm relative to bregma (Fig. 7c, d); 3) M1 (*p* < 0.0001), M2 (*p* = 0.001), Cg1 (*p* = 0.0416) and AI (*p* = 0.0004) at the level of − 2.1 mm relative to bregma (Fig. 7e, f); 4) S1 (*p* < 0.0001) and M1 (*p* < 0.0001) at the level of − 1.98 mm relative to bregma (Fig. 7g); 5) S1 (*p* < 0.0001) and M1 (*p* = 0.0107) at the level of − 1.7 mm relative to bregma (Fig. 7i). Compared to the vehicle + vehicle group, MA combined with *d*-fenfluramine (i.e. MA (1 mg/kg, i.p.) + *d*-fenfluramine (5 mg/kg, i.p.) treatment group) significantly increased c-*fos* expression in the: 1) FrA (*p* < 0.0001), PrL (*p* = 0.0275), LO (*p* = 0.0333) and DLO (*p* = 0.0009) at the level of − 2.68 mm relative to bregma (Fig. 7a, b); 2) M1 (*p* = 0.0002), M2 (*p* = 0.0001) and AI (*p* = 0.0011) at the level of − 2.34 mm relative to bregma (Fig. 7c, d); 3) M1 (*p* < 0.0001), M2 (*p* = 0.0082) and AI (*p* < 0.0001) at the level of − 2.1 mm relative to bregma (Fig. 7e, f); 4) S1 (*p* < 0.0001), M1 (*p* < 0.0001) and AI (*p* = 0.0075) at the level of − 1.98 mm relative to bregma (Fig. 7g, h); 5) S1 (*p* < 0.0001), M1 (*p* = 0.0002) and M2 (*p* = 0.0409) at the level of – 1.7 mm relative to bregma (Fig. 7i). Compared to the vehicle + *d*-fenfluramine (5 mg/kg, i.p.) group, pre-treatment of MA (i.e. MA (1 mg/kg, i.p.) + *d*-fenfluramine (5 mg/kg, i.p.) group) significantly increased c-*fos* expression in the: 1) FrA (*p* < 0.0001), LO (*p* = 0.0344) and DLO (*p* = 0.0006) at the level of − 2.68 mm relative to bregma (Fig. 7a, b); 2) M2 (*p* = 0.0024) at the level of − 2.34 mm relative to bregma (Fig. 7c); 3) M1 (*p* = 0.0001), M2 (*p* = 0.0477) and AI (*p* = 0.0253) at the level of − 2.1 mm relative to bregma (Fig. 7e, f); 4) S1 (*p* < 0.0001) and M1 (*p* = 0.0005) at the level of − 1.98 mm relative to bregma (Fig. 7g); 5) S1 (*p* < 0.0001) and M1 (*p* = 0.0139) at the level of − 1.7 mm relative to bregma (Fig. 7i). There were no significant differences between the comparison of the MA + vehicle *vs*. MA + *d*-fenfluramine group in the c-*fos* expression in these regions of mice PFC. The result indicates that MA significantly increased *d*-fenfluramine-induced c-*fos* expression in several regions of mice PFC.

## Discussion

In the present investigation we utilized the HTR in mice as a measure of postsynaptic serotonergic 5-HT_2A_ receptor function, and the immediate early gene c-*fos* expression as a measure of neuronal activity [47, 48], to address: (i) the ontogenic mechanisms by which the non-selective monoamine releaser MA suppresses the ability of the more selective serotonin releaser *d*-fenfluramine to evoke the HTR in mice across different ages, and (ii) whether MA pretreatment can alter *d*-fenfluramine-evoked c-*fos* expression in different regions of mice PFC where this intriguing behavior starts. The main results of this study are as follows: (i) intraperitoneal administration of a 5 mg/kg dose of *d*-fenfluramine tested across different ages (20-, 30- and 60-day old) produces a maximal mean frequency of HTRs at age 30 days; (ii) both MA and the selective 5-HT_2A_ receptor antagonist EMD 281014, dose-dependently suppressed *d*-fenfluramine-induced HTR across the discussed age-range; (iii) MA-evoked indirect activation of inhibitory serotonergic 5-HT_1A_- and adrenergic ɑ_2_- receptors which suppressed *d*-fenfluramine-induced HTR in 20- and 30- day old mice; (iv) blockade of 5-HT_2A_ receptors by EMD 281014 did not prevent *d*-fenfluramine-evoked c-*fos* expression in different regions of the PFC, but it significantly increased the evoked c-*fos* in one PFC region (PrL at – 2.68 mm); (v) MA alone increased c-*fos* expression in several different regions of the PFC; and (vi) relative to *d*-fenfluramine alone treatment group, addition of MA with *d*-fenfluramine significantly increased c-*fos* expression in some regions of the PFC.

### Ontogenic development of *d*-fenfluramine-evoked HTR in mice

Male albino ICR mice have been employed in our previously published studies [6, 8, 13]. One of our previous studies found there is no significant difference between males and females following induction of the HTR by the hallucinogens, such as, DOI [42]. For the continuity of the experiments, we continued with the use of male albino ICR mice in this study. Although hallucinogens such as DOI can also induce ear-scratch response (ESR; a rapid scratching movement of the head, neck, or lateral area by either hind limb), it is easy to identify both HTR and ESR behaviors simultaneously [42]. In addition, the dose of *d*-fenfluramine used to evoke the HTR (2.5 — 5 mg/kg, i.p) did not produce any other behavior in the current nor in our published studies [8, 22].

In a comprehensive behavioral study involving 7-, 14-, 18-, 22-, 35-, 42-, 63-, 120-, and 180-day old mice, we have demonstrated that developmental expression of the HTR behavior following administration of different doses of the 5-HT_2A/C_ receptor agonist DOI (0, 1 and 2.5 mg/kg, i.p.) begins between postnatal ages 14 – 18 days, reaches a maximum at day 28 days, and gradually subsides post 60 days of birth [42]. Along with these findings, published expression levels of 5-HT_2A_ receptor mRNA in the rat fetal brain seem to parallel 5-HT_2A_ binding sites, and both parameters increase (1300% and 800%, respectively) from embryonic day 17 to postnatal ages 5 – 13 days [49]. Although biochemically 5-HT_2A_ receptors appear to be fully functional on postnatal day 1 since they maximally potentiate 5-HT-stimulated phosphoinositide turnover in rat cortical slices [50], the ability of 5-HT_2A_ receptor to evoke HTRs in response to DOI in mice requires 14 – 18 days of further development. Furthermore, the maximal observed frequency of DOI-evoked HTR on day 28 subsequently decreases with increasing age. Published biochemical studies support the latter finding since several 5-HT_2A_ receptor parameters decrease during ageing in both animals and humans, including 5-HT_2A_ receptor number, mRNA expression, binding affinity, and sensitivity of its signal transduction mechanisms [50–53].

Depending upon which post-receptor signaling studied, DOI is considered as a direct potent partial to full agonist of 5-HT_2A_ receptors [1]. Unlike DOI, *d*-fenfluramine is mainly viewed as an indirect-acting non-selective agonist of all serotonergic receptors since it more selectively releases serotonin, which subsequently evokes the HTR via activation of 5-HT_2A_ receptors. The induction of *d*-fenfluramine-evoked HTR depends up on the viability of presynaptic machinery to synthesize, store and release 5-HT, as well as the efficiency of postsynaptic 5-HT_2A_-receptors coupled with the production of HTR (see above). Thus far, the maturation onset as well as ontogenic development of *d*-fenfluramine-evoke HTR have not been examined. Our current findings demonstrate that a maximally-effective dose of *d*-fenfluramine previously tested in 25 – 30 day old mice [8], can evoke the HTR in an age-dependent and bell-shaped manner with respective mean HTR frequencies of 7.9 (± 0.98), 19.4 (± 1.32), and 5.2 (± 0.87) in 20-, 30- and 60-day old mice. Moreover, these mean HTR frequencies are much smaller (8, 3, and 6.2 times) than those caused by a 1 mg/kg (i.p.) dose of DOI during corresponding ages [13]. In fact, it appears that during postnatal day 20 there is a sufficient *d*-fenfluramine-sensitive pool of releasable 5-HT that can evoke a small number of HTRs, which subsequently increases to induce a greater frequency of HTRs at age 30 days. Indeed, transmitter studies also demonstrate significant tissue levels of serotonin and its major metabolite 5-HIAA are present in the rat brain tissue on gestation day 15, which dramatically increase by 200% and 700% respectively, by gestation day 19 [54]. Furthermore, serotonin and 5-HIAA brain tissue levels continue to increase across postnatal days 1, 3, 5, 7, 15, 25 and reach a maximal value at 40 days, after which decline by day 90 [55]. The observed decrease in *d*-fenfluramine-evoked HTR on day 60 seems to be due several changes that occur during development, including: i) reductions in 5-HT_2A_ receptor affinity and density, ii) decrease in 5-HT_2A_ receptor interaction with its corresponding downstream signaling [42, 50–53], iii) reduced presynaptic release of a *d*-fenfluramine-sensitive pool of 5-HT [56, 57], and iv) increase in efficiency of the inhibitory 5-HT_1A_ receptor [7, 58], whose concomitant activation by *d*-fenfluramine-induced release of 5-HT can suppress the evoked HTR during development (see below).

### Pretreatment with a selective 5-HT_2A_ receptor antagonist (EMD 281014) or MA block *d*-fenfluramine-evoked HTR

The 5-HT_2_ receptor subfamily comprises of the 5-HT_2A_, 5-HT_2B_ and 5-HT_2C_ subtypes which exhibit 46 – 50% overall sequence identity [59]. Although the hallucinogen DOI has high affinity for all 5-HT_2_ binding sites and can directly activate them [1], it evokes the HTR in rodents via specific activation of postsynaptic 5-HT_2A_ receptors in the PFC across different ages [13]. In fact, nonselective (e.g., ketanserin) and very selective (e.g., EMD 281014) antagonists of 5-HT_2A_ receptors prevent the DOI-evoked behavior [1, 13]. Likewise, *d*-fenfluramine can produce the HTR behavior [8], but indirectly via release of serotonin, which subsequently activates serotonergic 5-HT_2A_ receptors. Indeed, pretreatment with highly-selective antagonists/inverse agonists of 5-HT_2A_ receptors such as M100907 can suppress *d*-fenfluramine-induced HTR, whereas potent and selective 5-HT_2C_ receptor antagonists/inverse agonists (e.g., SB 252084, SB 242084) do not affect the evoked behavior [60]. In the current study we demonstrate that another highly selective and potent 5-HT_2A_ receptor antagonist EMD 281014 (0.001 – 0.05 mg/kg, i.p.), can suppress *d*-fenfluramine-induced HTR in a dose-dependent manner across postnatal days 20, 30, and 60 with similar ID_50_ values. Furthermore, as in our previous study with the HTR inducer DOI [13], we now demonstrate that low doses of MA (0.1 – 5 mg/kg, i.p.) also attenuate *d*-fenfluramine-evoked HTR across the above discussed age-range with similar ID_50_ values in 20- [0.24 (0.14 – 0.04) mg/kg] and 30-day old mice [0.3 (0.21 − 0.42) mg/kg] which both are less than its ID_50_ in 60-day old mice [0.48 (0.23 – 0.81) mg/kg]. The larger doses of MA required to inhibit the *d*-fenfluramine-induced HTR in 60-day old mice probably reflects the above discussed changes in serotonin receptors parameters during ageing process.

Since MA lacks direct affinity for 5-HT_2A_ receptors [61], we performed the following inhibitory drug interaction studies to provide possible explanation(s) for the observed suppression of *d*-fenfluramine-evoked HTR frequency by MA.

### Indirect activation of serotonergic 5-HT_1A-_ and adrenergic ɑ_2_-receptors are involved in the suppressive effects of acute MA on *d*-fenfluramine-induced HTR

MA is a non-selective releaser of monoamines and therefore increases the synaptic concentration of 5-HT, NE, and DA in the corresponding nerve terminals in the PFC [62]. Although the evoked HTR is a simple response to observe, the behavior is rather complex to interpret since activation of several receptors can negatively or positively modulate it [1]. In the context of selective (e.g., sertraline) or nonselective (e.g., cocaine) monoamine reuptake blockers, concomitant indirect activation of inhibitory serotonergic 5-HT_1A_ and adrenergic a_2_-receptors play a prominent suppressive role on DOI- or *d*-fenfluramine-evoked HTRs [8, 40]. In fact, anatomically both inhibitory serotonergic 5-HT_1A_- and adrenergic ɑ_2_-receptors, as well as the stimulatory 5-HT_2A_ receptors, are expressed on pyramidal neurons and inhibitory interneurons throughout prefrontal cortical regions [63–69]. Moreover, 5-HT_1A_ receptors colocalize with 5-HT_2A_ receptors in the PFC, and stimulation of the former suppresses the in vivo biochemical, electrical, and behavioral effects of DOI on the latter receptors [7, 70–72]. However, during development the potent suppressive effect of the nonselective monoamine releaser MA on DOI-induced HTR, appears to be mainly dependent upon indirect activation of 5-HT_1A_ receptors rather additional involvement of adrenegic a_2_-recptors [13]. In the current study we questioned whether concomitant indirect stimulation of 5-HT_1A_- or adrenergic ɑ_2_-recptors by MA, would attenuate *d*-fenfluramine-induced HTRs.

Thus, we initially explored whether blockade of inhibitory 5-HT_1A_ receptors via the use of its selective antagonist WAY 100635, could reverse the inhibitory effect of MA on *d*-fenfluramine-induced HTR during development. We chose to confine our drug interaction studies in 20- and 30-day old mice since: i) 60-day old mice produced very few HTRs in response to a 5 mg/kg (i.p.) dose of *d*-fenfluramine, and ii) neither serotonergic 5-HT_1A_ nor adrenergic ɑ_2_-receptor blockade could reverse the inhibitory effect of MA on DOI-evoked HTRs in 60-day old mice [13]. In 30-day old mice, WAY 100635 (0.25 mg/kg, i.p.) pretreatment not only significantly increased the ability of *d*-fenfluramine to evoke a greater frequency of HTRs, but it also significantly reversed the inhibitory effect of MA (1 mg/kg, i.p.) on *d*-fenfluramine-induced HTR. A similar pattern of results was obtained in 20-day old mice, but the discussed increases in *d*-fenfluramine-evoked HTR failed to attain significance. It is interesting to note that although pretreatment with the same dose of WAY100635 failed to enhance the ability of DOI (1 mg/kg, i.p.) to evoke greater frequency of HTRs in 20-, 30-, or 60-day old mice, it did significantly reverse the suppressive effects of MA on DOI-induced HTRs in 20- and 30-day old mice [13]. These results suggest that basal brain serotonin levels do not affect the DOI-evoked HTR frequency when both WAY100635 and DOI are administered together intraperitoneally. However, when WAY100635 is directly injected in the PFC of rats, it can evoke the HTR by itself, and further potentiates the ability of centrally-administered DOI to induce HTR [12]. Furthermore, when administered peripherally, WAY100635 by itself can evoke the HTR in a 5-HT_2A_ antagonist (SR46349B)-sensitive manner when the behavior is measured within 5 h of the light cycle, and not during the dark cycle [73]. We then investigated whether ɑ_2_-adrenergic receptors could play a role in the inhibitory effect of MA on *d*-fenfluramine-induced HTR via use of its selective antagonist RS 79948. Pretreatment with RS 79948 (0.1 mg/kg, i.p.) significantly reversed the inhibitory effect of MA on *d*-fenfluramine-induced HTR in both 20-, and 30-day old mice. Interestingly, RS 79948 pretreatment could not significantly reverse the inhibitory effect of MA on the DOI-induced HTR in 20-, 30- or 60-day old mice [13]. The above discussed antagonist-interaction differences in DOI- and *d*-fenfluramine-evoked HTR during development could be due to the: i) choice of HTR inducer used in the two studies, and ii) different suppressive doses of MA were utilized in the two studies in that a 5 mg/kg dose of MA was used in the DOI-induced HTR study [13], whereas in the current study a 1 mg/kg dose of MA was used. These inhibitory 5-HT_1A_- and ɑ_2_-receptors’ functional interaction with the 5-HT_2A_ receptor is not confined to the effects of monoamine releasers and/ or reuptake blockers, but administration of direct-acting agonists of these receptors also demonstrate such functional interaction among these receptors [1, 7, 12]. Finally, our current and recent publication [13] demonstrate differential developmental interaction between inhibitory serotonergic 5-HT_1A_- and adrenergic ɑ_2_-receptors’ with the stimulatory 5-HT_2A_ receptors in the PFC during ageing which could be the basis why SSRIs in children can evoke suicidal tendencies (see introduction). Moreover, significant pharmacodynamic interactions occur following exposure to either selective (e.g., sertraline) or nonselective (e.g., cocaine) monoamine reuptake inhibitors, or monoamine releasers (e.g., 3,4-methylenedioxymethamphetamine (MDMA; ‘ecstasy’), which blunt the neuroendocrine effects of *d*-fenfluramine and 5-HT_1A_ receptor agonists in both humans and rodents [74–77]. Moreover, chronic SSRI use dampens response to MDMA-assisted therapy for post-traumatic stress disorder [78].

### Can blockade of 5-HT_2A_ receptors (EMD 281014) or stimulation (MA) of other receptors alter *d*-fenfluramine-evoked c-*fos* immunoreactivity in the mouse PFC?

We have previously demonstrated that blockade of 5-HT_2A_ receptor by EMD 281014 suppresses both DOI-evoked HTR behavior and c-*fos* immunoreactivity in different regions of the PFC [13]. In the current study, administration of *d*-fenfluramine caused significant expressions of c-*fos* in several but not all regions of mice PFC examined from rostral to caudal sections. This finding is in line with published immunohistochemical studies demonstrating Fos-like proteins are expressed rapidly and transiently in specific regions of the rat brain following a single administration of *d*-fenfluramine [79, 80]. Although in this study blockade of 5-HT_2A_ receptors by EMD 281014 did not prevent *d*-fenfluramine-evoked c-*fos* expression in any tested region of the PFC, it did significantly increase the evoked c-*fos* in the PrL at the level of bregma – 2.68 mm. The latter divergent finding is not unusual since the dopamine D_2_ receptor antagonist sulpiride has been shown to prevent MA-induced Fos expression in several regions of rodent brain except the striatum, where it dose-dependently increased MA-induced Fos-immunoreactivity [81]. Together with the fact that pretreatment with either 5-HT_1_- or 5-HT_2A/2C_- receptor antagonists do not attenuate *d*-fenfluramine-induced Fos-like protein expression in the brain [82, 83], these data suggest that other receptors are probably activated by *d*-fenfluramine administration, especially additional serotonergic receptors. On the other hand, although MA pretreatment could suppress the DOI-evoked HTR, it did significantly increase c-*fos* expression in several but not all regions of the PFC examined when administered either alone or in combination with DOI [13]. In the current study, MA also caused significant increases in c-*fos* expression in several regions of the PFC when administered alone, which is consistent with previous publications [13, 84, 85]. Furthermore, despite the inhibitory effect of MA on *d*-fenfluramine-induced HTR, inclusion of MA with *d*-fenfluramine caused additional c-*fos* expression in some but not all regions of the mouse PFC. These immunohistochemical findings suggest that additional receptor systems are probably activated by both *d*-fenfluramine and MA, especially the other serotonergic receptors. In fact, in vivo microdialysis findings indicate that *d*-fenfluramine and its metabolites, norfenfluramine, produce dose-related elevations of extracellular 5-HT, NE, and DA in rat frontal cortex [86].

## Conclusion

The current study has established the ontogenic development of *d*-fenfluramine-induced HTR in mice demonstrating maximal frequency of the behavior occurs at age 30 days. Pretreatment with ultra-low doses of EMD 281014 or low-doses of MA blocked *d*-fenfluramine-induced HTR in a dose-dependent manner across the age-range tested. The suppressive effects of MA on *d*-fenfluramine-evoked HTR during development are due to concomitant activation of inhibitory serotonergic 5-HT_1A_- and adrenergic ɑ_2_-receptors. Despite the inhibitory effect of EMD 281014 on *d*-fenfluramine-induced HTR, it failed to prevent *d*-fenfluramine-induced c-*fos* expression in the mouse PFC and increased the evoked c-*fos* in the PrL at the level of bregma-2.68 mm, which may be due to activation of other serotonergic receptors. Moreover, when administered alone, *d*-fenfluramine and MA by themselves significantly increased c-*fos* expression in several different regions of the PFC, and their combination caused significant additive increases in some specific regions, which is due to additional effects of MA either on 5-HT_2A_ receptor function or other monoaminergic receptor systems. These findings provide an ontogenic rationale for potential drug interactions as well as implications for the indirect effects of *d*-fenfluramine through differing release of monoamines across ageing which may differentially and concomitantly stimulate the inhibitory (i.e., 5-HT_1A_ and adrenergic α_2_ receptors) and stimulatory postsynaptic serotonergic 5-HT_2A_ receptors in the PFC which mediates *d*-fenfluramine-evoked HTR.

## Supplementary Information

**Additional file 1.** Raw data used for this publication.

## Data Availability

All data generated in this study and statistical methods used are included in Additional file 1—raw data.

## Abbreviations

AI: Agranular insular cortex
Cg1: Cingulate cortex area 1
CNS: Central nervous system
DA: Dopamine
DAT: DA transporter
DLO: Dorsal lateral orbital cortex
DOI: ( ±)− 2, 5-Dimethoxy-4-iodoamphetamine
DP: Dorsal peduncular cortex
FrA: Frontal associated cortex
HTR: Head-twitch response
IL: Infralimbic cortex
LC: Locus coeruleus
LO: Lateral orbital cortex
M1: Primary motor cortex
M2: Secondary motor cortex
MO: Medial orbital cortex
mPFC: The medial prefrontal cortex
NE: Norepinephrine
NET: NE transporter
PFC: Prefrontal cortex
PrL: Prelimbic cortex
RN: Raphe nuclei
S1: Primary somatosensory area
SERT: Serotonin transporter
TAAR1: Trace amine-associated receptor 1
VMAT-2: Vesicular monoamine transporter-2
VO: Ventral orbital cortex
VTA: Ventral tegmental area
