# Supplementary material for: Effects of low-doses of methamphetamine on d-fenfluramine-induced head-twitch response (HTR) in mice during ageing and c-fos expression in the prefrontal cortex
Source: BMC Neurosci. 2023 Jan 11;24:2. doi: 10.1186/s12868-022-00766-0 (PMC9835290; doi:10.1186/s12868-022-00766-0)
Supplement: Supplementary file 1 — Additional file 1. Raw data used for this publication. [file 12868_2022_766_MOESM1_ESM.docx]

**Table 1**

The frequency of HTR induced by *d*-fenfluramine (5 mg/kg, i.p.) across different ages in mice.

| **Number of mice** | **Frequency of HTR** | | |
| --- | --- | --- | --- |
|  | **20-day old mice** | **30-day old mice** | **60-day old mice** |
| 1 | 12 | 15 | 1 |
| 2 | 8 | 18 | 3 |
| 3 | 4 | 20 | 4 |
| 4 | 6 | 16 | 2 |
| 5 | 12 | 25 | 7 |
| 6 | 7 | 20 | 8 |
| 7 | 3 | 23 | 9 |
| 8 | 8 | 26 | 7 |
| 9 | 8 | 17 | 7 |
| 10 | 11 | 14 | 4 |

Raw data for figure 1. Statistical analysis: one-way ANOVA followed by Tukey's multiple comparisons test.

**Table 2**

Varying doses of the selective 5-HT_2A_ receptor antagonist EMD 281014 (0, 0.001, 0.005, 0.01 and 0.05 mg/kg, i.p.) suppressed the frequency of HTR induced by *d*-fenfluramine (5 mg/kg, i.p.) in 20-, 30- and 60-day old mice.

| **age** | **Frequency of HTR** | | | | | | | | |
| --- | --- | --- | --- | --- | --- | --- | --- | --- | --- |
|  |  | | **EMD (mg/kg, i.p.) + *d*-fenfluramine (5mg/kg, i.p.)** | | | | | |  |
|  | **n** | **Vehicle + vehicle** | | **0** | **0.001** | **0.005** | **0.01** | **0.05** | |
| **20-day old mice** | 1 | 0 | | 23 | 8 | 2 | 3 | 0 | |
|  | 2 | 0 | | 11 | 4 | 3 | 1 | 0 | |
|  | 3 | 0 | | 9 | 2 | 1 | 1 | 1 | |
|  | 4 | 0 | | 4 | 10 | 1 | 0 | 0 | |
|  | 5 | 0 | | 4 | 2 | 1 | 0 | 1 | |
|  | 6 | 0 | | 3 | 7 | 5 | 3 | 0 | |
|  | 7 |  | | 12 | 6 | 0 | 1 | 0 | |
|  | 8 |  | | 6 |  |  |  |  | |
| **30-day old mice** | 1 | 0 | | 20 | 12 | 5 | 7 | 0 | |
|  | 2 | 0 | | 23 | 12 | 12 | 5 | 1 | |
|  | 3 | 0 | | 26 | 18 | 8 | 7 | 1 | |
|  | 4 | 0 | | 17 | 18 | 6 | 3 | 1 | |
|  | 5 | 0 | | 32 | 15 | 3 | 4 | 0 | |
|  | 6 | 0 | | 14 | 11 | 6 | 3 |  | |
| **60-day old mice** | 1 | 0 | | 1 | 1 | 3 | 0 | 0 | |
|  | 2 | 0 | | 4 | 6 | 4 | 0 | 0 | |
|  | 3 | 0 | | 3 | 5 | 1 | 0 | 0 | |
|  | 4 | 0 | | 11 | 8 | 4 | 2 | 0 | |
|  | 5 | 0 | | 8 | 5 | 2 | 0 | 1 | |
|  | 6 | 0 | | 9 | 3 | 0 | 1 |  | |
|  | 7 |  | | 7 | 7 | 1 |  |  | |
|  | 8 |  | | 7 |  |  |  |  | |
|  | 9 |  | | 9 |  |  |  |  | |

Raw data for figure 2. Statistical analysis: two-way ANOVA followed by Dunnett's multiple comparisons test.

**Table 3**

Effects of varying doses of MA on the frequency of HTR induced by *d*-fenfluramine (5 mg/kg, i.p.) in 20 (MA at 0, 0.1, 0.25, 1 mg/kg, i.p.)-, 30 (MA at 0, 0.1, 0.25, 1, 2.5 mg/kg, i.p.)- and 60 (MA at 0, 1, 2.5, 5 mg/kg, i.p.)-day old mice.

| **Frequency of HTR** | | | | | | | |
| --- | --- | --- | --- | --- | --- | --- | --- |
| **20-day old mice** |  |  | **MA (mg/kg, i.p.) + *d*-fenfluramine (5mg/kg, i.p.)** | | | | |
|  | **n** | **Vehicle + vehicle** | **0** | **0.1** | **0.25** | **1** |  |
|  | 1 | 0 | 3 | 10 | 4 | 0 |  |
|  | 2 | 0 | 8 | 8 | 4 | 0 |  |
|  | 3 | 0 | 12 | 7 | 4 | 0 |  |
|  | 4 | 0 | 10 | 6 | 3 | 0 |  |
|  | 5 | 0 | 8 | 4 | 5 | 1 |  |
|  | 6 | 0 | 4 | 7 |  | 2 |  |
|  | 7 |  | 6 |  |  | 1 |  |
|  | 8 |  | 12 |  |  |  |  |
|  | 9 |  | 7 |  |  |  |  |
| **30-day old mice** |  |  | **MA (mg/kg, i.p.) + *d*-fenfluramine (5mg/kg, i.p.)** | | | | |
|  | **n** | **Vehicle + vehicle** | **0** | **0.1** | **0.25** | **1** | **2.5** |
|  | 1 | 0 | 15 | 17 | 12 | 7 | 0 |
|  | 2 | 0 | 20 | 16 | 9 | 1 | 1 |
|  | 3 | 0 | 16 | 17 | 14 | 1 | 0 |
|  | 4 | 0 | 19 | 16 | 17 | 4 | 2 |
|  | 5 | 0 | 18 | 10 | 7 | 4 | 1 |
|  | 6 | 0 | 20 | 12 | 10 | 3 |  |
|  | 7 |  | 16 |  |  |  |  |
|  | 8 |  | 25 |  |  |  |  |
|  | 9 |  | 17 |  |  |  |  |
| **60-day old mice** |  |  | **MA (mg/kg, i.p.) + *d*-fenfluramine (5mg/kg, i.p.)** | | | | |
|  | **n** | **Vehicle + vehicle** | **0** | **1** | **2.5** | **5** |  |
|  | 1 | 0 | 1 | 3 | 0 | 0 |  |
|  | 2 | 0 | 4 | 3 | 0 | 0 |  |
|  | 3 | 0 | 3 | 1 | 0 | 0 |  |
|  | 4 | 0 | 5 | 1 | 2 | 0 |  |
|  | 5 | 0 | 3 | 1 | 1 | 0 |  |
|  | 6 | 0 | 4 |  | 0 |  |  |
|  | 7 |  | 2 |  |  |  |  |
|  | 8 |  | 11 |  |  |  |  |
|  | 9 |  | 8 |  |  |  |  |

Raw data for figure 3. Statistical analysis: Kruskal-Wallis non-parametric one-way ANOVA followed by Dunn’s post hoc test.

**Table 4**

Pretreatment with the selective 5-HT_1A_ receptor antagonist WAY 100635 (0.25 mg/kg, i.p.) reversed the inhibitory action of MA (1 mg/kg, i.p.) on *d*-fenfluramine-induced (5 mg/kg, i.p.) HTR in 20- and 30-day old mice.

| **Frequency of HTR** | | | | | | |
| --- | --- | --- | --- | --- | --- | --- |
| **20-day old mice** | **n** | **Vehicle+Vehicle+Vehicle** | **Vehicle+**  **Vehicle+**  ***d*-fenfluramine(5)** | **MA (1)+**  **Vehicle+**  ***d*-fenfluramine(5)** | **Vehicle+**  **WAY (0.25) +**  ***d*-fenfluramine(5)** | **MA(1)+**  **WAY(0.25) +**  ***d*-fenfluramine(5)** |
|  | 1 | 0 | 11 | 0 | 5 | 9 |
|  | 2 | 0 | 12 | 0 | 9 | 6 |
|  | 3 | 0 | 11 | 0 | 10 | 7 |
|  | 4 | 0 | 9 | 0 | 11 | 7 |
|  | 5 | 0 | 4 | 1 | 12 | 6 |
|  | 6 | 0 | 4 | 2 | 19 | 6 |
|  | 7 |  | 3 | 1 | 10 | 6 |
|  | 8 |  | 12 |  |  |  |
|  | 9 |  | 6 |  |  |  |
| **30-day old mice** | **n** | **Vehicle+Vehicle+Vehicle** | **Vehicle+**  **Vehicle+**  ***d*-fenfluramine(5)** | **MA (1)+**  **Vehicle+**  ***d*-fenfluramine(5)** | **Vehicle+**  **WAY (0.25) +**  ***d*-fenfluramine(5)** | **MA(1)+**  **WAY(0.25) +**  ***d*-fenfluramine(5)** |
|  | 1 | 0 | 13 | 10 | 37 | 4 |
|  | 2 | 0 | 16 | 6 | 32 | 16 |
|  | 3 | 0 | 27 | 8 | 40 | 31 |
|  | 4 | 0 | 10 | 0 | 33 | 22 |
|  | 5 | 0 | 17 | 4 | 42 | 32 |
|  | 6 | 0 | 23 | 5 | 35 | 44 |
|  | 7 |  | 28 | 4 |  | 20 |
|  | 8 |  | 17 | 8 |  | 18 |
|  | 9 |  |  |  |  |  |

Raw data for figure 4a. Statistical analysis: two-way ANOVA followed by Dunnett's multiple comparisons test.

**Table 5**

Pretreatment with the selective ɑ_2_-adrenergic receptor antagonist RS 79948 (0.1 mg/kg, i.p.) reversed the inhibitory action of MA (1 mg/kg, i.p.) on *d*-fenfluramine-induced (5 mg/kg, i.p.) HTR in 20- and 30-day old mice.

| **Frequency of HTR** | | | | | | |
| --- | --- | --- | --- | --- | --- | --- |
| **20-day old mice** | **n** | **Vehicle+Vehicle+Vehicle** | **Vehicle+**  **Vehicle+**  ***d*-fenfluramine(5)** | **MA (1)+**  **Vehicle+**  ***d*-fenfluramine(5)** | **Vehicle+**  **RS (0.1) +**  ***d*-fenfluramine(5)** | **MA(1)+**  **RS (0.1) +**  ***d*-fenfluramine(5)** |
|  | 1 | 0 | 11 | 0 | 12 | 7 |
|  | 2 | 0 | 12 | 0 | 10 | 4 |
|  | 3 | 0 | 11 | 0 | 17 | 11 |
|  | 4 | 0 | 9 | 0 | 14 | 8 |
|  | 5 | 0 | 4 | 1 | 11 | 10 |
|  | 6 | 0 | 4 | 2 | 25 | 11 |
|  | 7 |  | 3 | 1 |  | 11 |
|  | 8 |  | 12 |  |  | 12 |
|  | 9 |  | 6 |  |  |  |
| **30-day old mice** | **n** | **Vehicle+Vehicle+Vehicle** | **Vehicle+**  **Vehicle+**  ***d*-fenfluramine(5)** | **MA (1)+**  **Vehicle+**  ***d*-fenfluramine(5)** | **Vehicle+**  **RS (0.1) +**  ***d*-fenfluramine(5)** | **MA(1)+**  **RS (0.1) +**  ***d*-fenfluramine(5)** |
|  | 1 | 0 | 13 | 10 | 26 | 32 |
|  | 2 | 0 | 16 | 6 | 27 | 13 |
|  | 3 | 0 | 27 | 8 | 23 | 12 |
|  | 4 | 0 | 10 | 0 | 19 | 16 |
|  | 5 | 0 | 17 | 4 | 7 | 11 |
|  | 6 | 0 | 23 | 5 | 26 | 22 |
|  | 7 |  | 28 | 4 | 36 | 22 |
|  | 8 |  | 17 | 8 |  |  |
|  | 9 |  |  |  |  |  |

Raw data for figure 4b. Statistical analysis: two-way ANOVA followed by Dunnett's multiple comparisons test.

**Table 6**

Effects of the selective 5-HT_2A_ receptor antagonist EMD 281014 on *d*-fenfluramine-induced c-*fos* expression in different regions at bregma -2.68 mm in the PFC of mice.

**Bregma -2.68 mm**

| **Vehicle +**  **Vehicle** | **Number of c-*fos*** | | | | | | |
| --- | --- | --- | --- | --- | --- | --- | --- |
|  | **n** | **FrA** | **PrL** | **MO** | **VO** | **LO** | **DLO** |
|  | 1 | 95 | 46 | 337 | 330 | 132 | 38 |
|  | 2 | 173 | 61 | 287 | 323 | 116 | 37 |
|  | 3 | 117 | 31 | 367 | 358 | 231 | 56 |
|  | 4 | 102 | 31 | 308 | 223 | 113 | 24 |
|  | 5 | 202 | 54 | 344 | 130 | 63 | 22 |
|  | 6 | 156 | 133 | 427 | 400 | 352 | 130 |
| **Vehicle +**  **d-fenfluramine** | **Number of c-*fos*** | | | | | | |
|  | **n** | **FrA** | **PrL** | **MO** | **VO** | **LO** | **DLO** |
|  | 1 | 264 | 28 | 248 | 140 | 84 | 53 |
|  | 2 | 343 | 60 | 204 | 337 | 164 | 51 |
|  | 3 | 322 | 73 | 224 | 200 | 84 | 33 |
|  | 4 | 380 | 92 | 373 | 301 | 163 | 63 |
|  | 5 | 659 | 104 | 394 | 383 | 204 | 100 |
|  | 6 | 487 | 65 | 301 | 198 | 194 | 95 |
| **EMD 281014 +**  **Vehicle** | **Number of c-*fos*** | | | | | | |
|  | **n** | **FrA** | **PrL** | **MO** | **VO** | **LO** | **DLO** |
|  | 1 | 22 | 8 | 48 | 323 | 12 | 2 |
|  | 2 | 218 | 37 | 167 | 150 | 101 | 57 |
|  | 3 | 319 | 46 | 150 | 181 | 181 | 80 |
|  | 4 | 222 | 33 | 129 | 133 | 137 | 85 |
|  | 5 | 294 | 17 | 256 | 106 | 136 | 78 |
|  | 6 | 192 | 56 | 298 | 155 | 166 | 91 |
| **EMD 281014 +**  **d-fenfluramine** | **Number of c-*fos*** | | | | | | |
|  | **n** | **FrA** | **PrL** | **MO** | **VO** | **LO** | **DLO** |
|  | 1 | 252 | 87 | 152 | 378 | 152 | 73 |
|  | 2 | 111 | 165 | 288 | 353 | 102 | 32 |
|  | 3 | 365 | 199 | 218 | 203 | 158 | 194 |
|  | 4 | 460 | 151 | 190 | 465 | 125 | 14 |
|  | 5 | 787 | 76 | 196 | 107 | 99 | 31 |
|  | 6 | 740 | 89 | 340 | 316 | 321 | 133 |

Raw data for figure 6 a, b. Statistical analysis: One-way analysis of variance (ANOVA) followed by Tukey's multiple test.

**Table 7**

Effects of the selective 5-HT_2A_ receptor antagonist EMD 281014 on *d*-fenfluramine-induced c-*fos* expression in different regions at bregma -2.34 mm in the PFC of mice.

**Bregma -2.34 mm**

| **Vehicle +**  **Vehicle** | **Number of c-*fos*** | | | | | | | | |
| --- | --- | --- | --- | --- | --- | --- | --- | --- | --- |
|  | **n** | **M1** | **M2** | **Cg1** | **PrL** | **MO** | **VO** | **LO** | **AI** |
|  | 1 | 66 | 51 | 57 | 70 | 166 | 197 | 129 | 52 |
|  | 2 | 34 | 236 | 83 | 152 | 138 | 191 | 218 | 113 |
|  | 3 | 32 | 286 | 137 | 335 | 241 | 373 | 393 | 103 |
|  | 4 | 89 | 200 | 51 | 155 | 235 | 276 | 269 | 77 |
|  | 5 | 68 | 322 | 43 | 198 | 278 | 230 | 343 | 101 |
|  | 6 | 24 | 290 | 129 | 215 | 235 | 274 | 285 | 95 |
| **Vehicle +**  **d-fenfluramine** | **Number of c-*fos*** | | | | | | | | |
|  | **n** | **M1** | **M2** | **Cg1** | **PrL** | **MO** | **VO** | **LO** | **AI** |
|  | 1 | 165 | 351 | 62 | 145 | 350 | 468 | 299 | 186 |
|  | 2 | 110 | 344 | 88 | 118 | 172 | 318 | 167 | 309 |
|  | 3 | 135 | 447 | 103 | 141 | 237 | 325 | 243 | 290 |
|  | 4 | 182 | 497 | 118 | 177 | 291 | 344 | 244 | 144 |
|  | 5 | 215 | 679 | 138 | 192 | 276 | 352 | 223 | 179 |
|  | 6 | 263 | 660 | 52 | 131 | 350 | 392 | 356 | 253 |
| **EMD 281014 +**  **Vehicle** | **Number of c-*fos*** | | | | | | | | |
|  | **n** | **M1** | **M2** | **Cg1** | **PrL** | **MO** | **VO** | **LO** | **AI** |
|  | 1 | 54 | 167 | 50 | 75 | 189 | 197 | 89 | 56 |
|  | 2 | 70 | 213 | 52 | 108 | 233 | 284 | 138 | 63 |
|  | 3 | 199 | 624 | 93 | 131 | 227 | 347 | 288 | 245 |
|  | 4 | 101 | 383 | 100 | 119 | 223 | 207 | 192 | 106 |
|  | 5 | 94 | 339 | 44 | 114 | 314 | 296 | 171 | 95 |
|  | 6 | 32 | 91 | 32 | 101 | 229 | 168 | 153 | 72 |
| **EMD 281014 +**  **d-fenfluramine** | **Number of c-*fos*** | | | | | | | | |
|  | **n** | **M1** | **M2** | **Cg1** | **PrL** | **MO** | **VO** | **LO** | **AI** |
|  | 1 | 179 | 337 | 142 | 186 | 248 | 302 | 118 | 330 |
|  | 2 | 102 | 494 | 145 | 180 | 322 | 495 | 181 | 444 |
|  | 3 | 384 | 215 | 74 | 263 | 190 | 306 | 417 | 280 |
|  | 4 | 208 | 297 | 69 | 188 | 372 | 483 | 162 | 204 |
|  | 5 | 275 | 873 | 94 | 105 | 314 | 309 | 259 | 149 |
|  | 6 | 282 | 943 | 80 | 147 | 278 | 302 | 397 | 257 |

Raw data for figure 6 c, d. Statistical analysis: One-way analysis of variance (ANOVA) followed by Tukey's multiple test.

**Table 8**

Effects of the selective 5-HT_2A_ receptor antagonist EMD 281014 on *d*-fenfluramine-induced c-*fos* expression in different regions at bregma -2.1 mm in the PFC of mice.

**Bregma -2.1 mm**

| **Vehicle +**  **Vehicle** | **Number of c-*fos*** | | | | | | | | |
| --- | --- | --- | --- | --- | --- | --- | --- | --- | --- |
|  | **n** | **M1** | **M2** | **Cg1** | **PrL** | **MO** | **VO** | **LO** | **AI** |
|  | 1 | 69 | 114 | 96 | 136 | 169 | 284 | 154 | 107 |
|  | 2 | 207 | 308 | 136 | 181 | 174 | 407 | 188 | 197 |
|  | 3 | 162 | 407 | 155 | 237 | 394 | 206 | 331 | 118 |
|  | 4 | 94 | 315 | 147 | 187 | 177 | 174 | 206 | 115 |
|  | 5 | 273 | 425 | 184 | 270 | 283 | 278 | 189 | 183 |
|  | 6 | 128 | 399 | 179 | 187 | 299 | 251 | 316 | 177 |
| **Vehicle +**  **d-fenfluramine** | **Number of c-*fos*** | | | | | | | | |
|  | **n** | **M1** | **M2** | **Cg1** | **PrL** | **MO** | **VO** | **LO** | **AI** |
|  | 1 | 337 | 285 | 121 | 120 | 199 | 490 | 242 | 238 |
|  | 2 | 361 | 485 | 119 | 113 | 136 | 315 | 128 | 197 |
|  | 3 | 401 | 334 | 85 | 77 | 114 | 243 | 143 | 165 |
|  | 4 | 486 | 327 | 127 | 142 | 206 | 286 | 245 | 293 |
|  | 5 | 668 | 845 | 198 | 165 | 238 | 378 | 234 | 374 |
|  | 6 | 1020 | 738 | 188 | 187 | 283 | 353 | 329 | 345 |
| **EMD 281014 +**  **Vehicle** | **Number of c-*fos*** | | | | | | | | |
|  | **n** | **M1** | **M2** | **Cg1** | **PrL** | **MO** | **VO** | **LO** | **AI** |
|  | 1 | 82 | 108 | 131 | 28 | 53 | 131 | 47 | 45 |
|  | 2 | 213 | 274 | 84 | 115 | 297 | 342 | 196 | 165 |
|  | 3 | 408 | 457 | 151 | 168 | 273 | 326 | 161 | 254 |
|  | 4 | 404 | 462 | 114 | 90 | 146 | 277 | 183 | 203 |
|  | 5 | 404 | 255 | 185 | 123 | 255 | 379 | 202 | 286 |
|  | 6 | 317 | 227 | 73 | 123 | 165 | 309 | 194 | 214 |
| **EMD 281014 +**  **d-fenfluramine** | **Number of c-*fos*** | | | | | | | | |
|  | **n** | **M1** | **M2** | **Cg1** | **PrL** | **MO** | **VO** | **LO** | **AI** |
|  | 1 | 344 | 770 | 244 | 274 | 299 | 659 | 153 | 262 |
|  | 2 | 165 | 705 | 184 | 225 | 443 | 415 | 311 | 299 |
|  | 3 | 361 | 417 | 152 | 269 | 337 | 445 | 256 | 211 |
|  | 4 | 518 | 472 | 182 | 106 | 154 | 308 | 330 | 298 |
|  | 5 | 328 | 611 | 167 | 140 | 163 | 305 | 223 | 378 |
|  | 6 | 972 | 600 | 84 | 96 | 209 | 248 | 240 | 246 |

Raw data for figure 6 e, f. Statistical analysis: One-way analysis of variance (ANOVA) followed by Tukey's multiple test.

**Table 9**

Effects of the selective 5-HT_2A_ receptor antagonist EMD 281014 on *d*-fenfluramine-induced c-*fos* expression in different regions at bregma -1.98 mm in the PFC of mice.

**Bregma -1.98 mm**

| **Vehicle +**  **Vehicle** | **Number of c-*fos*** | | | | | | | | | | |
| --- | --- | --- | --- | --- | --- | --- | --- | --- | --- | --- | --- |
|  | **n** | **S1** | **M1** | **M2** | **Cg1** | **PrL** | **IL** | **MO** | **VO** | **LO** | **AI** |
|  | 1 | 89 | 88 | 193 | 114 | 271 | 134 | 92 | 94 | 265 | 120 |
|  | 2 | 50 | 100 | 198 | 125 | 288 | 130 | 91 | 169 | 273 | 171 |
|  | 3 | 52 | 107 | 909 | 337 | 257 | 190 | 214 | 108 | 148 | 313 |
|  | 4 | 133 | 118 | 353 | 196 | 148 | 124 | 118 | 248 | 318 | 160 |
|  | 5 | 128 | 140 | 294 | 150 | 214 | 241 | 174 | 201 | 211 | 239 |
|  | 6 | 51 | 121 | 295 | 190 | 303 | 205 | 213 | 343 | 225 | 145 |
| **Vehicle +**  **d-fenfluramine** | **Number of c-*fos*** | | | | | | | | | | |
|  | **n** | **S1** | **M1** | **M2** | **Cg1** | **PrL** | **IL** | **MO** | **VO** | **LO** | **AI** |
|  | 1 | 348 | 213 | 288 | 156 | 297 | 131 | 130 | 194 | 289 | 273 |
|  | 2 | 241 | 234 | 473 | 205 | 282 | 130 | 124 | 240 | 255 | 301 |
|  | 3 | 127 | 200 | 290 | 134 | 180 | 83 | 90 | 178 | 158 | 149 |
|  | 4 | 392 | 434 | 417 | 140 | 219 | 84 | 85 | 197 | 299 | 372 |
|  | 5 | 411 | 424 | 511 | 189 | 332 | 145 | 124 | 184 | 212 | 331 |
|  | 6 | 395 | 397 | 249 | 104 | 214 | 164 | 182 | 232 | 420 | 426 |
| **EMD 281014 +**  **Vehicle** | **Number of c-*fos*** | | | | | | | | | | |
|  | **n** | **S1** | **M1** | **M2** | **Cg1** | **PrL** | **IL** | **MO** | **VO** | **LO** | **AI** |
|  | 1 | 45 | 59 | 56 | 43 | 92 | 60 | 58 | 41 | 263 | 67 |
|  | 2 | 79 | 179 | 329 | 171 | 240 | 119 | 116 | 134 | 368 | 153 |
|  | 3 | 246 | 323 | 330 | 116 | 236 | 208 | 182 | 201 | 239 | 226 |
|  | 4 | 220 | 331 | 275 | 121 | 160 | 107 | 123 | 183 | 228 | 286 |
|  | 5 | 235 | 393 | 498 | 217 | 269 | 110 | 111 | 120 | 298 | 378 |
|  | 6 | 103 | 243 | 275 | 56 | 98 | 42 | 51 | 198 | 352 | 222 |
| **EMD 281014 +**  **d-fenfluramine** | **Number of c-*fos*** | | | | | | | | | | |
|  | **n** | **S1** | **M1** | **M2** | **Cg1** | **PrL** | **IL** | **MO** | **VO** | **LO** | **AI** |
|  | 1 | 340 | 286 | 291 | 226 | 249 | 208 | 222 | 233 | 295 | 389 |
|  | 2 | 378 | 331 | 292 | 187 | 231 | 103 | 160 | 211 | 245 | 278 |
|  | 3 | 274 | 334 | 497 | 210 | 347 | 139 | 133 | 242 | 210 | 306 |
|  | 4 | 446 | 468 | 364 | 166 | 171 | 141 | 162 | 198 | 220 | 383 |
|  | 5 | 351 | 275 | 340 | 162 | 210 | 104 | 151 | 142 | 224 | 437 |
|  | 6 | 280 | 406 | 298 | 98 | 161 | 96 | 132 | 156 | 411 | 309 |

Raw data for figure 6 g, h. Statistical analysis: One-way analysis of variance (ANOVA) followed by Tukey's multiple test.

**Table 10**

Effects of the selective 5-HT_2A_ receptor antagonist EMD 281014 on *d*-fenfluramine-induced c-*fos* expression in different regions at bregma -1.7 mm in the PFC of mice.

**Bregma -1.7 mm**

| **Vehicle +**  **Vehicle** | **Number of c-*fos*** | | | | | | | |
| --- | --- | --- | --- | --- | --- | --- | --- | --- |
|  | **n** | **S1** | **M1** | **M2** | **Cg1** | **PrL** | **IL** | **DP** |
|  | 1 | 59 | 91 | 233 | 151 | 232 | 176 | 76 |
|  | 2 | 136 | 144 | 264 | 165 | 213 | 241 | 99 |
|  | 3 | 52 | 276 | 825 | 303 | 166 | 142 | 173 |
|  | 4 | 92 | 179 | 461 | 221 | 262 | 227 | 115 |
|  | 5 | 179 | 297 | 496 | 236 | 161 | 120 | 179 |
|  | 6 | 67 | 128 | 426 | 227 | 103 | 219 | 117 |
| **Vehicle +**  **d-fenfluramine** | **Number of c-*fos*** | | | | | | | |
|  | **n** | **S1** | **M1** | **M2** | **Cg1** | **PrL** | **IL** | **DP** |
|  | 1 | 376 | 193 | 248 | 179 | 231 | 180 | 103 |
|  | 2 | 201 | 311 | 505 | 197 | 145 | 198 | 103 |
|  | 3 | 220 | 437 | 501 | 194 | 127 | 194 | 112 |
|  | 4 | 282 | 639 | 562 | 193 | 156 | 83 | 108 |
|  | 5 | 244 | 299 | 422 | 149 | 143 | 108 | 99 |
|  | 6 | 446 | 277 | 314 | 172 | 223 | 170 | 84 |
| **EMD 281014 +**  **Vehicle** | **Number of c-*fos*** | | | | | | | |
|  | **n** | **S1** | **M1** | **M2** | **Cg1** | **PrL** | **IL** | **DP** |
|  | 1 | 47 | 58 | 126 | 69 | 81 | 81 | 56 |
|  | 2 | 101 | 228 | 256 | 176 | 183 | 133 | 138 |
|  | 3 | 297 | 399 | 360 | 207 | 231 | 158 | 114 |
|  | 4 | 223 | 249 | 292 | 182 | 240 | 120 | 127 |
|  | 5 | 468 | 441 | 294 | 217 | 261 | 164 | 104 |
|  | 6 | 148 | 241 | 279 | 97 | 131 | 106 | 70 |
| **EMD 281014 +**  **d-fenfluramine** | **Number of c-*fos*** | | | | | | | |
|  | **n** | **S1** | **M1** | **M2** | **Cg1** | **PrL** | **IL** | **DP** |
|  | 1 | 262 | 372 | 270 | 218 | 193 | 118 | 162 |
|  | 2 | 391 | 207 | 357 | 159 | 110 | 102 | 98 |
|  | 3 | 241 | 282 | 330 | 234 | 195 | 108 | 159 |
|  | 4 | 207 | 351 | 282 | 143 | 101 | 107 | 102 |
|  | 5 | 311 | 290 | 280 | 181 | 202 | 146 | 103 |
|  | 6 | 249 | 327 | 338 | 111 | 87 | 257 | 65 |

Raw data for figure 6 i, j. Statistical analysis: One-way analysis of variance (ANOVA) followed by Tukey's multiple test.

**Table 11**

Effects of MA on *d*-fenfluramine-induced c-*fos* expression in different regions at bregma -2.68 mm in the PFC of mice.

**Bregma -2.68 mm**

| **Vehicle +**  **Vehicle** | **Number of c-*fos*** | | | | | | |
| --- | --- | --- | --- | --- | --- | --- | --- |
|  | **n** | **FrA** | **PrL** | **MO** | **VO** | **LO** | **DLO** |
|  | 1 | 97 | 11 | 148 | 150 | 73 | 21 |
|  | 2 | 90 | 15 | 208 | 184 | 61 | 15 |
|  | 3 | 107 | 45 | 203 | 268 | 82 | 24 |
|  | 4 | 109 | 66 | 179 | 366 | 181 | 50 |
|  | 5 | 58 | 12 | 399 | 442 | 202 | 60 |
|  | 6 | 171 | 63 | 561 | 328 | 325 | 116 |
| **Vehicle +**  **d-fenfluramine** | **Number of c-*fos*** | | | | | | |
|  | **n** | **FrA** | **PrL** | **MO** | **VO** | **LO** | **DLO** |
|  | 1 | 232 | 32 | 148 | 109 | 99 | 27 |
|  | 2 | 256 | 31 | 191 | 185 | 136 | 42 |
|  | 3 | 329 | 56 | 275 | 280 | 210 | 63 |
|  | 4 | 225 | 63 | 274 | 193 | 160 | 20 |
|  | 5 | 317 | 47 | 206 | 61 | 101 | 43 |
|  | 6 | 401 | 105 | 321 | 341 | 222 | 65 |
| **MA +**  **Vehicle** | **Number of c-*fos*** | | | | | | |
|  | **n** | **FrA** | **PrL** | **MO** | **VO** | **LO** | **DLO** |
|  | 1 | 663 | 101 | 356 | 370 | 317 | 124 |
|  | 2 | 685 | 105 | 319 | 388 | 404 | 136 |
|  | 3 | 357 | 52 | 436 | 188 | 330 | 196 |
|  | 4 | 610 | 151 | 440 | 240 | 241 | 98 |
|  | 5 | 458 | 95 | 322 | 364 | 295 | 200 |
|  | 6 | 369 | 72 | 397 | 439 | 318 | 194 |
| **MA +**  **d-fenfluramine** | **Number of c-*fos*** | | | | | | |
|  | **n** | **FrA** | **PrL** | **MO** | **VO** | **LO** | **DLO** |
|  | 1 | 917 | 150 | 437 | 441 | 386 | 223 |
|  | 2 | 535 | 86 | 342 | 164 | 227 | 128 |
|  | 3 | 824 | 65 | 243 | 213 | 316 | 167 |
|  | 4 | 568 | 92 | 300 | 307 | 250 | 80 |
|  | 5 | 651 | 146 | 528 | 359 | 351 | 155 |
|  | 6 | 650 | 35 | 358 | 235 | 174 | 146 |

Raw data for figure 7 a, b. Statistical analysis: One-way analysis of variance (ANOVA) followed by Tukey's multiple test.

**Table 12**

Effects of MA on *d*-fenfluramine-induced c-*fos* expression in different regions at bregma -2.34 mm in the PFC of mice.

**Bregma -2.34 mm**

| **Vehicle +**  **Vehicle** | **Number of c-*fos*** | | | | | | | | |
| --- | --- | --- | --- | --- | --- | --- | --- | --- | --- |
|  | **n** | **M1** | **M2** | **Cg1** | **PrL** | **MO** | **VO** | **LO** | **AI** |
|  | 1 | 42 | 50 | 29 | 106 | 198 | 250 | 118 | 28 |
|  | 2 | 8 | 141 | 31 | 90 | 309 | 310 | 125 | 52 |
|  | 3 | 92 | 234 | 42 | 85 | 140 | 268 | 164 | 123 |
|  | 4 | 48 | 213 | 69 | 139 | 157 | 420 | 296 | 116 |
|  | 5 | 58 | 107 | 68 | 135 | 304 | 453 | 466 | 72 |
|  | 6 | 72 | 462 | 72 | 159 | 496 | 698 | 450 | 84 |
| **Vehicle +**  **d-fenfluramine** | **Number of c-*fos*** | | | | | | | | |
|  | **n** | **M1** | **M2** | **Cg1** | **PrL** | **MO** | **VO** | **LO** | **AI** |
|  | 1 | 272 | 446 | 84 | 152 | 413 | 414 | 415 | 272 |
|  | 2 | 158 | 268 | 35 | 95 | 309 | 298 | 324 | 181 |
|  | 3 | 133 | 388 | 64 | 144 | 339 | 446 | 441 | 224 |
|  | 4 | 59 | 196 | 59 | 109 | 295 | 372 | 264 | 182 |
|  | 5 | 182 | 250 | 51 | 138 | 457 | 314 | 351 | 173 |
|  | 6 | 187 | 250 | 85 | 155 | 381 | 399 | 305 | 174 |
| **MA +**  **Vehicle** | **Number of c-*fos*** | | | | | | | | |
|  | **n** | **M1** | **M2** | **Cg1** | **PrL** | **MO** | **VO** | **LO** | **AI** |
|  | 1 | 107 | 535 | 95 | 169 | 265 | 527 | 377 | 327 |
|  | 2 | 217 | 641 | 54 | 140 | 356 | 498 | 336 | 233 |
|  | 3 | 242 | 405 | 90 | 185 | 300 | 421 | 500 | 254 |
|  | 4 | 172 | 507 | 151 | 161 | 252 | 471 | 322 | 207 |
|  | 5 | 196 | 435 | 138 | 137 | 245 | 439 | 651 | 246 |
|  | 6 | 165 | 558 | 111 | 251 | 286 | 340 | 388 | 244 |
| **MA +**  **d-fenfluramine** | **Number of c-*fos*** | | | | | | | | |
|  | **n** | **M1** | **M2** | **Cg1** | **PrL** | **MO** | **VO** | **LO** | **AI** |
|  | 1 | 298 | 900 | 156 | 197 | 307 | 471 | 431 | 348 |
|  | 2 | 217 | 490 | 93 | 173 | 412 | 332 | 361 | 250 |
|  | 3 | 260 | 770 | 42 | 100 | 321 | 424 | 453 | 282 |
|  | 4 | 102 | 450 | 64 | 72 | 248 | 426 | 280 | 73 |
|  | 5 | 202 | 604 | 139 | 261 | 329 | 421 | 441 | 169 |
|  | 6 | 297 | 496 | 82 | 172 | 374 | 345 | 514 | 323 |

Raw data for figure 7 c, d. Statistical analysis: One-way analysis of variance (ANOVA) followed by Tukey's multiple test.

**Table 13**

Effects of MA on *d*-fenfluramine-induced c-*fos* expression in different regions at bregma -2.1 mm in the PFC of mice.

**Bregma -2.1 mm**

| **Vehicle +**  **Vehicle** | **Number of c-*fos*** | | | | | | | | |
| --- | --- | --- | --- | --- | --- | --- | --- | --- | --- |
|  | **n** | **M1** | **M2** | **Cg1** | **PrL** | **MO** | **VO** | **LO** | **AI** |
|  | 1 | 55 | 155 | 106 | 172 | 210 | 436 | 150 | 56 |
|  | 2 | 71 | 218 | 133 | 154 | 196 | 569 | 215 | 87 |
|  | 3 | 204 | 263 | 131 | 134 | 167 | 329 | 201 | 145 |
|  | 4 | 87 | 182 | 98 | 131 | 153 | 283 | 168 | 85 |
|  | 5 | 83 | 294 | 156 | 194 | 246 | 732 | 424 | 153 |
|  | 6 | 126 | 557 | 135 | 169 | 323 | 800 | 434 | 183 |
| **Vehicle +**  **d-fenfluramine** | **Number of c-*fos*** | | | | | | | | |
|  | **n** | **M1** | **M2** | **Cg1** | **PrL** | **MO** | **VO** | **LO** | **AI** |
|  | 1 | 352 | 245 | 117 | 165 | 390 | 528 | 342 | 354 |
|  | 2 | 300 | 329 | 85 | 119 | 264 | 454 | 241 | 271 |
|  | 3 | 538 | 370 | 167 | 258 | 316 | 675 | 330 | 396 |
|  | 4 | 482 | 236 | 80 | 114 | 218 | 443 | 219 | 148 |
|  | 5 | 406 | 402 | 137 | 160 | 320 | 447 | 315 | 248 |
|  | 6 | 356 | 404 | 142 | 176 | 261 | 368 | 216 | 259 |
| **MA +**  **Vehicle** | **Number of c-*fos*** | | | | | | | | |
|  | **n** | **M1** | **M2** | **Cg1** | **PrL** | **MO** | **VO** | **LO** | **AI** |
|  | 1 | 750 | 547 | 152 | 187 | 240 | 683 | 225 | 493 |
|  | 2 | 899 | 492 | 151 | 151 | 274 | 403 | 396 | 493 |
|  | 3 | 544 | 707 | 186 | 216 | 238 | 306 | 249 | 329 |
|  | 4 | 885 | 498 | 196 | 136 | 300 | 366 | 382 | 313 |
|  | 5 | 701 | 522 | 207 | 175 | 206 | 500 | 275 | 304 |
|  | 6 | 672 | 681 | 220 | 131 | 232 | 435 | 299 | 375 |
| **MA +**  **d-fenfluramine** | **Number of c-*fos*** | | | | | | | | |
|  | **n** | **M1** | **M2** | **Cg1** | **PrL** | **MO** | **VO** | **LO** | **AI** |
|  | 1 | 1178 | 708 | 210 | 177 | 183 | 468 | 272 | 601 |
|  | 2 | 633 | 336 | 90 | 100 | 204 | 529 | 413 | 447 |
|  | 3 | 916 | 515 | 95 | 176 | 286 | 469 | 289 | 423 |
|  | 4 | 629 | 558 | 103 | 112 | 222 | 307 | 254 | 231 |
|  | 5 | 733 | 478 | 174 | 161 | 239 | 391 | 355 | 417 |
|  | 6 | 992 | 490 | 100 | 151 | 217 | 417 | 369 | 570 |

Raw data for figure 7 e, f. Statistical analysis: One-way analysis of variance (ANOVA) followed by Tukey's multiple test.

**Table 14**

Effects of MA on *d*-fenfluramine-induced c-*fos* expression in different regions at bregma -1.98 mm in the PFC of mice.

**Bregma -1.98 mm**

| **Vehicle +**  **Vehicle** | **Number of c-*fos*** | | | | | | | | | | |
| --- | --- | --- | --- | --- | --- | --- | --- | --- | --- | --- | --- |
|  | **n** | **S1** | **M1** | **M2** | **Cg1** | **PrL** | **IL** | **MO** | **VO** | **LO** | **AI** |
|  | 1 | 18 | 25 | 109 | 102 | 222 | 179 | 124 | 315 | 234 | 69 |
|  | 2 | 49 | 50 | 158 | 99 | 190 | 110 | 109 | 339 | 359 | 159 |
|  | 3 | 29 | 60 | 140 | 82 | 192 | 140 | 99 | 176 | 188 | 81 |
|  | 4 | 34 | 76 | 223 | 109 | 225 | 115 | 75 | 44 | 176 | 111 |
|  | 5 | 48 | 96 | 364 | 194 | 320 | 159 | 131 | 186 | 444 | 226 |
|  | 6 | 98 | 109 | 520 | 169 | 343 | 195 | 224 | 488 | 533 | 381 |
| **Vehicle +**  **d-fenfluramine** | **Number of c-*fos*** | | | | | | | | | | |
|  | **n** | **S1** | **M1** | **M2** | **Cg1** | **PrL** | **IL** | **MO** | **VO** | **LO** | **AI** |
|  | 1 | 275 | 268 | 603 | 185 | 398 | 200 | 195 | 274 | 427 | 472 |
|  | 2 | 193 | 196 | 299 | 128 | 251 | 160 | 199 | 262 | 300 | 322 |
|  | 3 | 240 | 287 | 500 | 209 | 449 | 187 | 196 | 305 | 415 | 358 |
|  | 4 | 85 | 290 | 230 | 157 | 217 | 132 | 139 | 174 | 219 | 194 |
|  | 5 | 196 | 202 | 337 | 131 | 250 | 134 | 117 | 240 | 455 | 364 |
|  | 6 | 202 | 265 | 382 | 260 | 373 | 117 | 96 | 114 | 243 | 186 |
| **MA +**  **Vehicle** | **Number of c-*fos*** | | | | | | | | | | |
|  | **n** | **S1** | **M1** | **M2** | **Cg1** | **PrL** | **IL** | **MO** | **VO** | **LO** | **AI** |
|  | 1 | 522 | 399 | 394 | 161 | 280 | 151 | 233 | 277 | 468 | 311 |
|  | 2 | 290 | 566 | 324 | 144 | 284 | 203 | 114 | 206 | 307 | 302 |
|  | 3 | 244 | 302 | 500 | 138 | 225 | 129 | 111 | 115 | 446 | 467 |
|  | 4 | 360 | 333 | 387 | 153 | 453 | 200 | 269 | 174 | 274 | 391 |
|  | 5 | 498 | 483 | 571 | 171 | 265 | 174 | 130 | 301 | 326 | 297 |
|  | 6 | 500 | 377 | 569 | 219 | 272 | 112 | 133 | 477 | 355 | 300 |
| **MA +**  **d-fenfluramine** | **Number of c-*fos*** | | | | | | | | | | |
|  | **n** | **S1** | **M1** | **M2** | **Cg1** | **PrL** | **IL** | **MO** | **VO** | **LO** | **AI** |
|  | 1 | 428 | 605 | 734 | 218 | 352 | 133 | 158 | 238 | 387 | 403 |
|  | 2 | 589 | 731 | 429 | 162 | 236 | 114 | 132 | 231 | 493 | 577 |
|  | 3 | 475 | 690 | 416 | 175 | 213 | 194 | 107 | 153 | 332 | 530 |
|  | 4 | 309 | 351 | 387 | 84 | 185 | 175 | 124 | 117 | 222 | 152 |
|  | 5 | 647 | 304 | 419 | 151 | 159 | 97 | 140 | 111 | 302 | 449 |
|  | 6 | 569 | 581 | 291 | 115 | 193 | 94 | 91 | 111 | 298 | 383 |

Raw data for figure 7 g, h. Statistical analysis: One-way analysis of variance (ANOVA) followed by Tukey's multiple test.

**Table 15**

Effects of MA on *d*-fenfluramine-induced c-*fos* expression in different regions at bregma -1.7 mm in the PFC of mice.

**Bregma -1.7 mm**

| **Vehicle +**  **Vehicle** | **Number of c-*fos*** | | | | | | | |
| --- | --- | --- | --- | --- | --- | --- | --- | --- |
|  | **n** | **S1** | **M1** | **M2** | **Cg1** | **PrL** | **IL** | **DP** |
|  | 1 | 22 | 44 | 212 | 211 | 262 | 220 | 137 |
|  | 2 | 38 | 118 | 198 | 147 | 216 | 201 | 125 |
|  | 3 | 56 | 95 | 222 | 122 | 133 | 137 | 111 |
|  | 4 | 72 | 79 | 180 | 174 | 174 | 128 | 99 |
|  | 5 | 70 | 116 | 463 | 261 | 294 | 179 | 181 |
|  | 6 | 94 | 182 | 617 | 302 | 366 | 204 | 165 |
| **Vehicle +**  **d-fenfluramine** | **Number of c-*fos*** | | | | | | | |
|  | **n** | **S1** | **M1** | **M2** | **Cg1** | **PrL** | **IL** | **DP** |
|  | 1 | 208 | 405 | 425 | 212 | 244 | 252 | 106 |
|  | 2 | 334 | 180 | 391 | 189 | 237 | 193 | 106 |
|  | 3 | 240 | 429 | 588 | 266 | 262 | 191 | 132 |
|  | 4 | 329 | 132 | 268 | 185 | 200 | 155 | 97 |
|  | 5 | 165 | 251 | 550 | 200 | 206 | 194 | 100 |
|  | 6 | 153 | 193 | 455 | 172 | 199 | 257 | 84 |
| **MA +**  **Vehicle** | **Number of c-*fos*** | | | | | | | |
|  | **n** | **S1** | **M1** | **M2** | **Cg1** | **PrL** | **IL** | **DP** |
|  | 1 | 599 | 309 | 437 | 239 | 173 | 269 | 99 |
|  | 2 | 396 | 363 | 475 | 243 | 119 | 220 | 76 |
|  | 3 | 429 | 469 | 578 | 216 | 329 | 226 | 98 |
|  | 4 | 659 | 324 | 495 | 224 | 139 | 159 | 172 |
|  | 5 | 518 | 429 | 401 | 146 | 345 | 265 | 120 |
|  | 6 | 542 | 584 | 595 | 235 | 220 | 147 | 150 |
| **MA +**  **d-fenfluramine** | **Number of c-*fos*** | | | | | | | |
|  | **n** | **S1** | **M1** | **M2** | **Cg1** | **PrL** | **IL** | **DP** |
|  | 1 | 409 | 451 | 576 | 144 | 162 | 291 | 158 |
|  | 2 | 655 | 996 | 678 | 185 | 222 | 128 | 141 |
|  | 3 | 655 | 680 | 519 | 166 | 201 | 107 | 143 |
|  | 4 | 422 | 280 | 314 | 117 | 93 | 272 | 85 |
|  | 5 | 724 | 413 | 643 | 138 | 100 | 307 | 76 |
|  | 6 | 504 | 552 | 481 | 197 | 123 | 102 | 87 |

Raw data for figure 7 i, j. Statistical analysis: One-way analysis of variance (ANOVA) followed by Tukey's multiple test.
